# Macrocarpal I induces immunogenic cell death and synergizes with immune checkpoint inhibition by targeting tubulin and PARP1 in colorectal cancer

**DOI:** 10.1038/s41420-025-02360-9

**Published:** 2025-02-22

**Authors:** Yaxin Zhang, Huali Li, Yali Zhao, Lingtao Liu, Yi Zhou, Xingyan Pan, Yanqing Ding, Wenting Liao, Lu Qi, Chengmei Huang, Na Tang

**Affiliations:** 1 State Key Laboratory of Oncology in South China, Guangdong Provincial Clinical Research Center for Cancer, Sun Yat-sen University Cancer Center, Guangzhou, 510060 P. R. China; 2 Department of Pathology, Shenzhen Longgang Central Hospital, Shenzhen, 518100 China; 3 Department of Pathology, School of Basic Medical Sciences, Southern Medical University, Guangzhou, 510515 China; 4 Department of Pathology, Shenzhen People’s Hospital, The Second Clinical Medical College, Jinan University, Shenzhen, 518020 China

**Keywords:** Drug development, Immunization

## Abstract

Colorectal cancer (CRC) presents an obstacle to immunotherapy, primarily because most cases are microsatellite stable (MSS) tumors, which are often described as “cold tumors” with limited immunogenicity. Recent studies have indicated that several therapeutic approaches, such as chemotherapy and targeted therapies, can elicit immunogenic cell death (ICD) and stimulate immune responses. However, challenges such as target affinity and in vivo pharmacokinetics limit the efficacy and immune response of current targeted therapies. In this study, we demonstrate that Macrocarpal I is a potent inducer of ICD by activating the PERK/eIF2A/ATF4/CHOP signaling pathway. Furthermore, Macrocarpal I induces apoptosis and ferroptosis, both of which act as triggers for ICD. Mechanistically, Macrocarpal I directly targets TUBB2B and PARP1, disrupting microtubule polymerization and DNA repair processes. Importantly, treatment with Macrocarpal I enhances the anti-tumor immune response and augments responsiveness to anti-PD-1 therapy in an MC38 syngeneic mouse model of CRC.

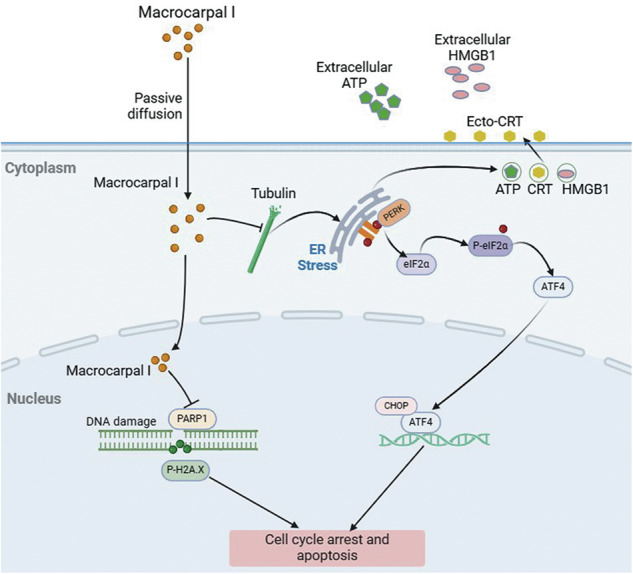

## Introduction

Colorectal cancer (CRC) is the third most common cancer globally and represents the second leading cause of cancer-related deaths worldwide [1]. Recent advances in primary and adjuvant therapies have improved the prognosis for CRC patients. However, the five-year survival rate for patients with metastatic CRC is a mere 12% [2]. The mortality rate in advanced CRC is primarily attributed to a combination of factors, including the limited availability of treatment options, persistent resistance to therapy, and notable toxicity associated with existing treatments. Recently, immune checkpoint inhibitors (ICIs) have demonstrated clinical efficacy in treating advanced solid tumors, such as melanoma, non-small cell lung cancer, renal cell carcinoma, and mismatch repair-deficient CRC [3, 4]. However, an estimated 80%-85% of CRC patients, harboring microsatellite stable (MSS) subtype tumors, exhibit a poor response to ICI therapy [5]. Therefore, developing effective and well-tolerated combinatorial strategies is crucial.

The insufficient infiltration of anti-tumor T cells is a primary reason for resistance to ICI therapy [6]. Immunogenic cell death (ICD) represents a form of cell death where dying cancer cells emit damage-associated molecular patterns (DAMPs) that subsequently boost antigen-presenting cell functionality and activate T cells [7]. ICD is marked by the membrane translocation of calreticulin (CRT) and the release of high mobility group protein B1 (HMGB1), ATP, heat shock proteins (HSPs), and ANXA1, all of which play roles in amplifying immune responses against tumor cells [8]. Recent literature suggests that various treatment strategies, such as chemotherapy, radiation therapy, and targeted therapies, are known to induce ICD and elicit both innate and adaptive immune responses [9–11]. However, challenges such as target affinity, in vivo pharmacokinetics, and deep niche localization limit the efficacy and immune response of current targeted therapies.

Naturally derived compounds, appealing for their low toxicity and cost, are considered potential immunoadjuvants due to their ability to trigger cancer stress responses and DAMPs via diverse mechanisms [12, 13]. Macrocarpal I, a natural compound, falls within the category of organic compounds known as lignans. Specifically, it is classified as a dibenzylbutyrolactone lignan that exhibits various biological activities, such as antioxidant and antimicrobial properties [14]. Previously, we demonstrated that Macrocarpal I effectively inhibited cell proliferation in vitro and reduced tumor growth in CRC cells in nude mice. Our findings revealed that Macrocarpal I treatment effectively induced apoptosis and disrupted the cytoskeleton in CRC cells [15]. Intriguingly, RNA-seq analysis showed that Macrocarpal I treatment altered the DNA mismatch repair signaling pathway-related gene expression [15]. However, the impact of Macrocarpal I on the anti-tumor immune response remains to be elucidated. In this study, we demonstrate that Macrocarpal I treatment induces ICD, reversing anti-PD-1 resistance and enhancing the anti-tumor immune response in CRC. Furthermore, we identified β-tubulin and PARP1 as effective targets for Macrocarpal I in CRC.

## Results

### Macrocarpal I induces immunogenic cell death in CRC cells

**Fig. 1:**
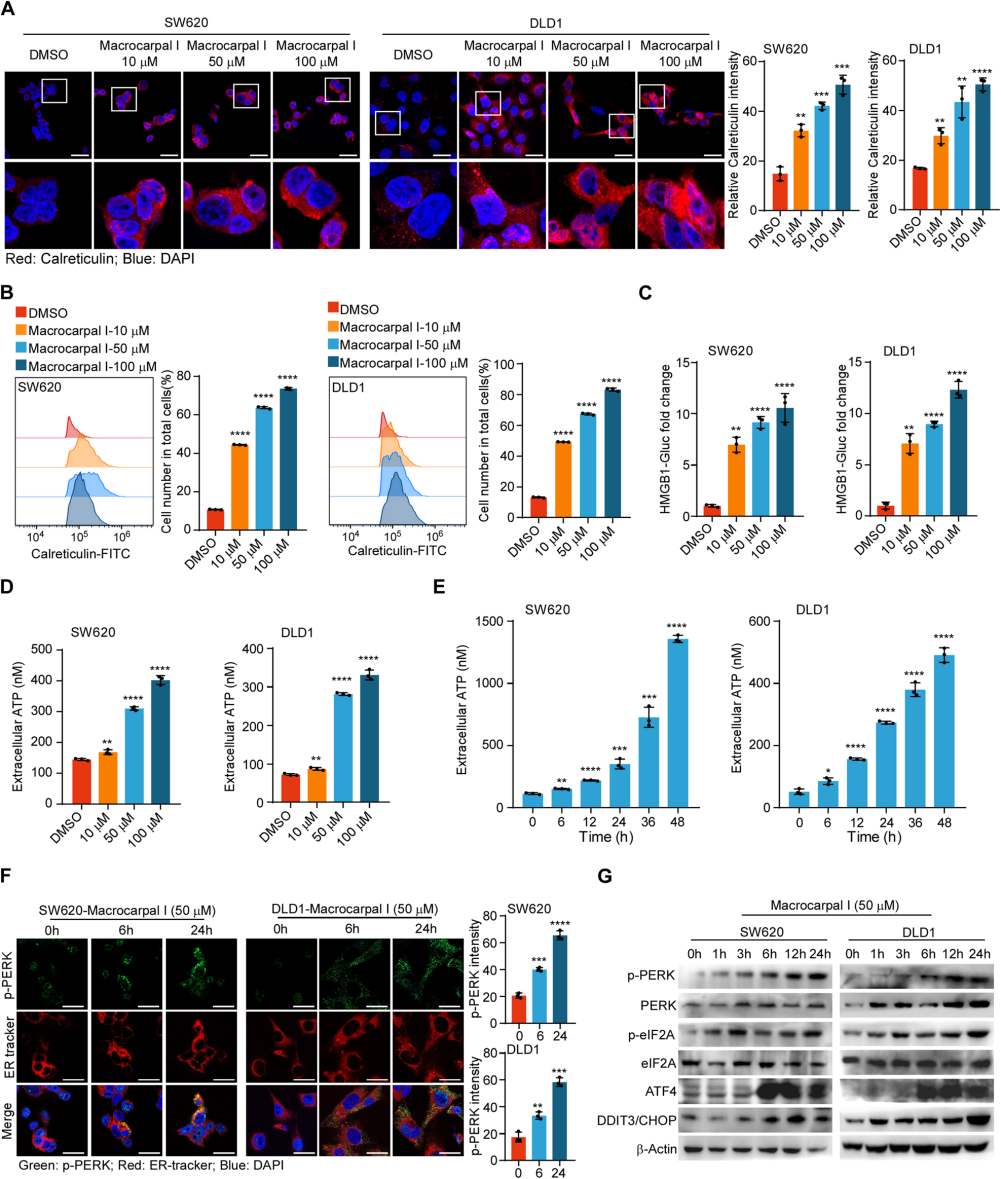
Macrocarpal I induces immunogenic cell death in CRC cells. **A** Immunofluorescence was utilized to detect the accumulation of calreticulin in SW620 and DLD1 cells treated with either DMSO or Macrocarpal I. Scale bar, 50 μm. **B** The expression levels of cell surface calreticulin, in response to DMSO or Macrocarpal I treatment, were measured using flow cytometry on SW620 and DLD1 cells. **C** The activity of the HMGB1-Gluc reporter in response to either DMSO or Macrocarpal I was meticulously analyzed within SW620 and DLD1 cells. **D**, **E** The chemiluminescence assay was utilized to detect ATP secretion in cell supernatants. **F** Representative confocal microscopy images of endogenous p-PERK clusters and ER visualized by IF staining of p-PERK (green) along with the ER tracker (red) in cells treated with Macrocarpal I at 50 μM for the indicated time intervals. The nucleus was stained with DAPI (blue). Scale bar, 50 μm. **G** The expression of PERK, p-PERK, eIF2A, p-eIF2A, ATF4, and CHOP was detected by western blotting. In A, B, C, D, E, mean ± SD, *n* = 3, two-tailed *t*-test, *p < 0.05, **p < 0.01, ***p < 0.001, ****p < 0.0001.

To evaluate the effect of Macrocarpal I on the immune response, we first measured the expression of ICD-associated DAMPs (CRT, HMGB1, and ATP) in CRC cell lines SW620 and DLD1. CRT exposure was increased in a dose-dependent manner upon Macrocarpal I treatment, as measured by IF and flow cytometry analyses (Fig. 1A, B; Fig. S1). The release of HMGB1 and ATP detected in cell culture supernatants also showed a dose-dependent effect after treatment (Fig. 1C, D). Furthermore, time-sequential analysis of extracellular ATP release showed a time-dependent increase over 0 to 48 h under 50 μM treatment (Fig. 1E). Since ICD is a stress-induced response where ER stress-related unfolded protein response (UPR) induces cell apoptosis [16], we examined the effect of Macrocarpal I on ER morphology and stress signaling. By using ER tracker dye, no morphological changes of ER were observed under Macrocarpal I, but there were significant aggregates of p-PERK (Fig. 1F). Western blot results also identified the escalated level of phosphorylated PERK, eIF2a, ATF4, and DDIT3/CHOP over time (Fig. 1G). These findings indicate that Macrocarpal I is a potent inducer of ICD, activating the PERK/eIF2A/ATF4/CHOP signaling pathway.

### Macrocarpal I triggers apoptosis and ferroptosis in CRC cells

**Fig. 2:**
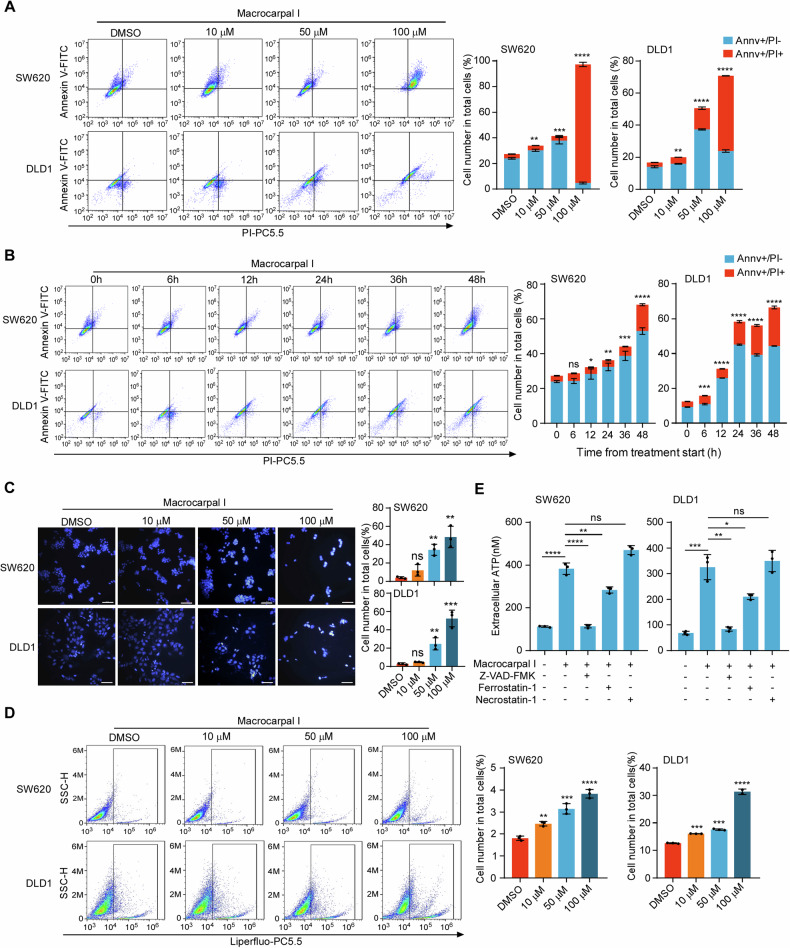
Macrocarpal I triggers apoptosis and ferroptosis in CRC cells. **A** Flow cytometry analysis of SW620 and DLD1 cells stained with Annexin V and PI after treatment with different concentrations of Macrocarpal I (10, 50, and 100 μM). The dot plots and quantification data represent Annexin V^+^/PI^-^ (early-stage apoptosis) and Annexin V^+^/PI^+^ (late-stage apoptosis) cells. **B** Flow cytometry analysis of SW620 and DLD1 cells stained with Annexin V/PI after treatment with Macrocarpal I at a concentration of 50 μM for 0 to 48 h. The representative quantified data show Annexin V^+^/PI^-^ and Annexin V^+^/PI^+^ cells. **C** Immunofluorescence detection of Hoechst 33342-stained SW620 and DLD1 cells treated with 50 μM of Macrocarpal I. Scale bar, 50 μm. **D** Flow cytometry analysis of liperfluo expression levels in SW620 and DLD1 cells treated with DMSO or Macrocarpal I. **E** Chemiluminescence assay detection of ATP secretion in cell supernatant from SW620 and DLD1 cells following treatment with Macrocarpal I (50 μM) in combination with Z-VAD-FMK, Ferrostatin-1 and Necrostatin-1. In A, B, C, D, E, mean ± SD, *n* = 3, two-tailed t-test, ns, no significance, *p < 0.05, **p < 0.01, ***p < 0.001, ****p < 0.0001.

Accumulating evidence has indicated that dying cells play a crucial role in immune response regulation by releasing or exposing DAMPs and thus acting as stimuli for ICD [17]. We then investigated the effect of Macrocarpal I on cell death pathways, including apoptosis, necroptosis, ferroptosis, pyroptosis, and senescence. The results showed that SW620 and DLD1 cells underwent early-stage apoptosis (Annexin-V^+^/PI^-^) in response to Macrocarpal I treatment at 50 μM, while late-stage apoptosis (Annexin-V^+^/PI^+^) was observed at 100 μM (Fig. 2A). After 24 h of Macrocarpal I treatment, the early-stage apoptotic populations increased with time in both CRC cell lines, while the late-stage populations increased in the subsequent 24 h (Fig. 2B). Next, apoptosis was characterized by staining with Hoechst 33342, showing that nuclear condensation was significantly increased after Macrocarpal I treatment (Fig. 2C). These data suggest that Macrocarpal I induces cell apoptosis. Moreover, ferroptosis analysis, characterized by lipid peroxidation, revealed Macrocarpal I had subtle effects on ferroptosis (Fig. 2D). Importantly, the apoptosis inhibitor Z-VAD-FMK completely abolished the extracellular secretion of ATP induced by Macrocarpal I. In addition, ferrostatin-1 (a ferroptosis inhibitor) also slightly reversed this effect (Fig. 2E; Fig. S2B–F). However, Necrostatin-1 (an inhibitor of necroptosis) had no effects on Macrocarpal I-induced ICD response (Fig. 2E; Fig. S2B, C), and nor did it influence senescence (Fig. S2A). In addition, we examined the cytotoxic effects of Macrocarpal I on non-tumor cells and found that the apoptosis of normal intestinal epithelial cells and immune cells remained unchanged after Macrocarpal I treatment (Fig. S2G). These results indicate that Macrocarpal I induces apoptosis and ferroptosis in tumor cells.

### Identification of TUBB2B and PARP1 as targets of Macrocarpal I

**Fig. 3:**
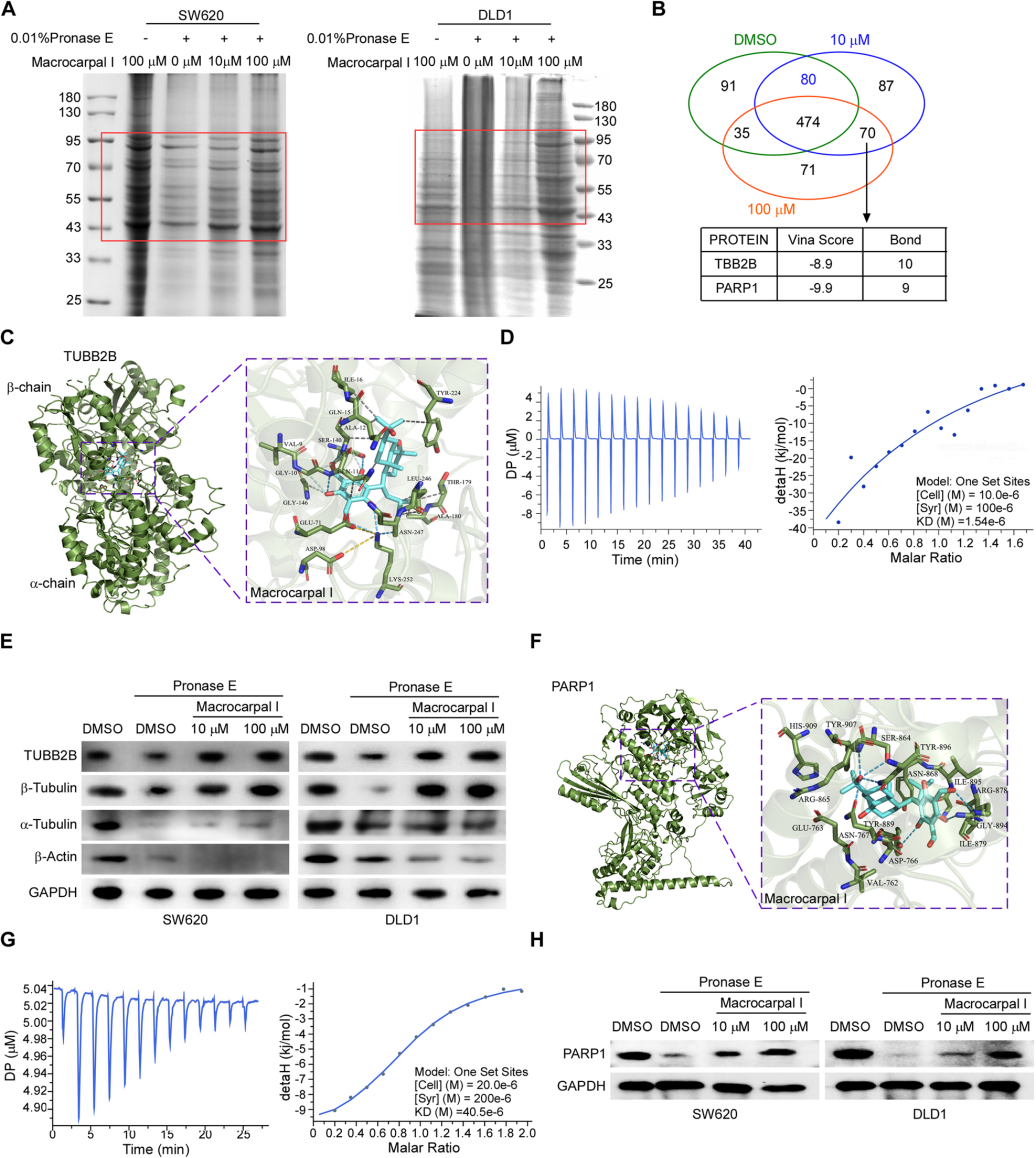
Identification of TUBB2B and PARP1 as targets of Macrocarpal I. **A** DARTS assay was conducted in the lysate of SW620 and DLD1 cells, with the presence of Macrocarpal I at the indicated concentration. **B** Overlapping results of mass spectrometry analysis showing statistical significance (*p* < 0.01 and coverage ≥2). **C** 3D illustration of the interaction between Macrocarpal I and TUBB2B, with the active site and amino acid residues labeled in purple, hydrogen bonds in blue, ionic interactions in gold and hydrophobic interactions in grey. **D** Isothermal titration curves show the binding of Macrocarpal I to TUBB2B. **E** DARTS assay measures the expression of TUBB2B upon treatment with Macrocarpal I in SW620 and DLD1 cells. **F** 3D illustration of the interaction between Macrocarpal I and PARP1, with the active site and amino acid residues labeled in purple, hydrogen bonds in blue, ionic interactions in gold and hydrophobic interactions in grey. **G** Isothermal titration curves showing the binding of PARP1 and Macrocarpal I. **H** DARTS assay measures the increase in PARP1 levels upon treatment with Macrocarpal I in SW620 and DLD1 cells.

To identify potential targets by which Macrocarpal I induces cell death and immune responses, we conducted Drug Affinity Responsive Target Stability (DARTS) assays in SW620 and DLD1 cells. Even after pronase E digestion, gel samples continued to exhibit protein bands (Fig. 3A). Mass spectrometry analysis identified Tubulin Beta 2B Class IIb (TUBB2B) and Poly (ADP-Ribose) Polymerase 1 (PARP1) as two significant candidates (Fig. 3B; Fig. S3A, B). PARP1 is an FDA-approved drug target [18]. TUBB2B encodes one of the isotypes of β-tubulin and constitutes a significant component of microtubules, which are the primary target of anticancer tubulin-binding agents [19].

Autodocking analysis revealed that the active pocket of Macrocarpal I towards TUBB2B is located at coordinates 25, -3, 4, with docking dimensions of 22, 22, 22 (Fig. 3C). Interestingly, Macrocarpal I shares a binding site with Colchicine at the junction of the α-chain and β-chain within the molecule (Fig. 3C; Fig. S3C). In contrast, Paclitaxel, which has been used in treating ovarian, non-small cell lung, and breast cancers for decades, displayed a distinct docking site on the edge of the β-chain, corresponding to Macrocarpal I (Fig. S3D).

To determine the effects of Macrocarpal I on cellular microtubules, his-tagged TUBB2B fusion protein was expressed in E. coli, subsequently purified using affinity and molecular sieve purification techniques. The binding affinity of Macrocarpal I for TUBB2B was quantified using isothermal titration calorimetry (ITC). The calculated dissociation constant for Macrocarpal I and TUBB2B was 1.54 × 10^-6 M, indicating a strong binding affinity (Fig. 3D; Fig. S3E). DARTS analysis revealed that the binding affinity diminished following digestion with 0.01% pronase E compared to the group treated with Macrocarpal I. A similar trend was observed with total β-tubulin, but there was minimal impact on α-tubulin and β-actin (Fig. 3E).

Additionally, PARP1, an FDA-approved drug target [18], was identified as a potential secondary target of Macrocarpal I. Autodocking analysis demonstrated that the active pocket of Macrocarpal I for PARP1 is 7, 9, and -12, with docking sizes of 28, 22, and 22, respectively (Fig. 3F). The binding affinity of Macrocarpal I for PARP1 was assessed using ITC and corrected the acronym to DARTS (Fig. 3G, H; Fig. S3F). Furthermore, PARP1 enzyme activity was significantly reduced after Macrocarpal I treatment in vitro (Fig. S3G). These findings suggest that Macrocarpal I could potentially be utilized as a dual-targeted small molecule drug.

### Macrocarpal I interferes with the polymerization of microtubules and DNA repair processes by inhibiting TUBB2B and PARP1

**Fig. 4:**
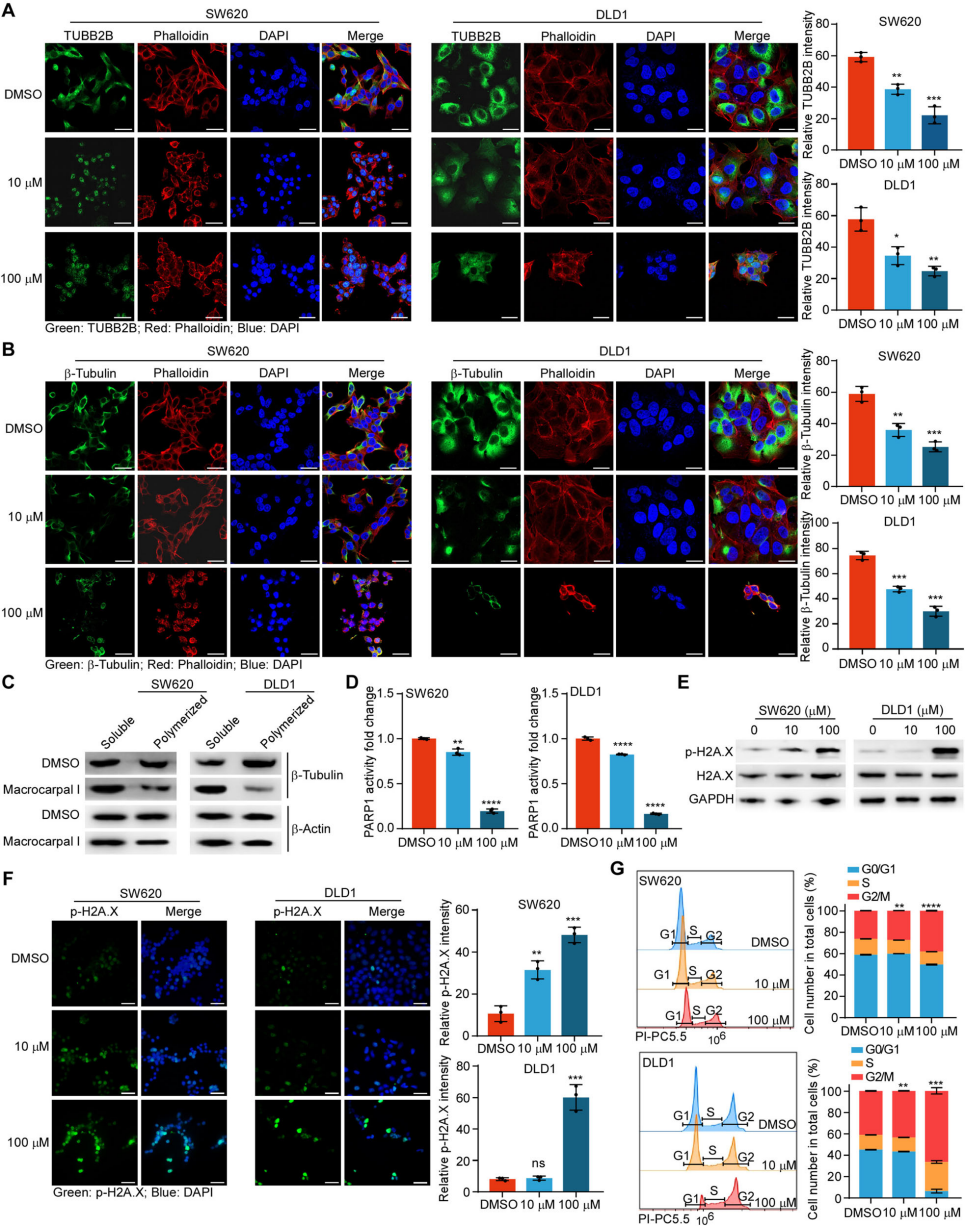
Macrocarpal I interferes with the polymerization of microtubules and DNA repair processes by inhibiting TUBB2B and PARP1. **A** SW620 and DLD1 cells were treated with the indicated drug concentrations for 24 h, and the expression of TUBB2B was examined using immunofluorescence. Scale bar, 50 μm. **B** Macrocarpal I altered the morphology of CRC cell cytoskeleton by detecting β‐tubulin. Scale bar, 50 μm. **C** SW620 and DLD1 cells were treated with the indicated drug concentrations for 24 h. The free and polymerized tubulin (soluble (S) and polymerized (P)) were separated and detected by western blotting. **D** ELISA analysis of PARP1 activity in SW620 and DLD1 cells. **E** Phosphorylation of H2A.X was examined by western blotting. **F** Immunofluorescence detection of p-H2A.X-stained SW620 and DLD1 cells treated with Macrocarpal I. Scale bar, 50 μm. **G** SW620 and DLD1 cells were treated with different concentrations of Macrocarpal I (0, 10, and 100 μM). The cell cycle phase distribution was analyzed by flow cytometry, showing the representative plots and quantification data for G0/G1, S and G2/M-phase cells. In A, B, D, F, G, mean ± SD, two-tailed *t*-test, ns, no significance, **p < 0.01, ***p < 0.001, ****p < 0.0001.

Given the established binding affinity with TUBB2B, we investigated the impact of Macrocarpal I on microtubule structure. We evaluated microtubule structure using anti-TUBB2B, anti-β-tubulin, and anti-α-tubulin staining in SW620 and DLD1 cells. Cells treated with Macrocarpal I at a concentration of 10 µM showed significantly reduced and fragmented TUBB2B and β-tubulin microtubule networks (Fig. 4A, B). Notably, the abundance of α-tubulin was unaffected by Macrocarpal I at this concentration (Fig. S4A), indicating a direct targeting of β-tubulin rather than α-tubulin. Ultracentrifugation showed that pellets from cells treated with Macrocarpal I exhibited substantially decreased levels of polymerized tubulin in a dose-dependent manner, corresponding to an increase in soluble tubulin in the supernatant (Fig. 4C), suggesting that Macrocarpal I promotes the transition of cellular microtubules from a polymerized state to a free state.

PARP is a nuclear enzyme crucial for repairing single-strand DNA breaks, and inhibiting PARP1 proves lethal in a broad spectrum of cancer cells. PARP1 enzyme activity was significantly reduced after Macrocarpal I treatment in SW620 and DLD1 cells (Fig. 4D). Western blot and immunofluorescence showed that Macrocarpal I treatment significantly increased the phosphorylation of H2A.X (Fig. 4E, F), a marker for double-strand DNA breaks and is indicative of PARP1 inhibition [20]. This result suggests that Macrocarpal I disrupts DNA repair processes. Considering the ability of both microtubule-interfering agents and PARP1 inhibitors to induce G2/M arrest and cell apoptosis [21], the effects of Macrocarpal I on the cell cycle were analyzed. Macrocarpal I (0, 10, and 100 μM) significantly increased G2/M phased cell ratio (Fig. 4G). Collectively, these findings suggest that Macrocarpal I interacts with TUBB2B and PARP1, disrupting microtubule polymerization and DNA repair processes, ultimately leading to an accumulation of DNA double-strand breaks and G2/M phase arrest.

To further elucidate the roles of TUBB2B and PARP1 in inducing ICD and cell death. TUBB2B and PARP1 were stably knocked down in CRC cells (Fig. S4B). It was found that the ER morphology did not change with TUBB2B interference, but significant aggregates of p-PERK were observed (Fig. S4C). The release of ATP and HMGB1 detected in cell culture supernatants also increased after TUBB2B knockdown (Fig. S4D and S4F). Flow cytometry analysis showed enhanced membrane translocation of CRT induced by TUBB2B knockdown (Fig. S4H). However, the interference with PARP1 did not activate the ICD response (Fig. S4E, S4G and S4I). We next investigated the impact of TUBB2B and PARP1 on cell death pathways. The results showed that CRC cells underwent apoptosis and ferroptosis in response to TUBB2B knockdown, but the knockdown of PARP1 only induced cell apoptosis (Fig. S4J, K). These results indicate that TUBB2B and PARP1 play crucial roles in inducing cell death, further confirming the potential of Macrocarpal I as a dual-target small molecule drug.

### Macrocarpal I promotes anti-tumor immune response in CRC

**Fig. 5:**
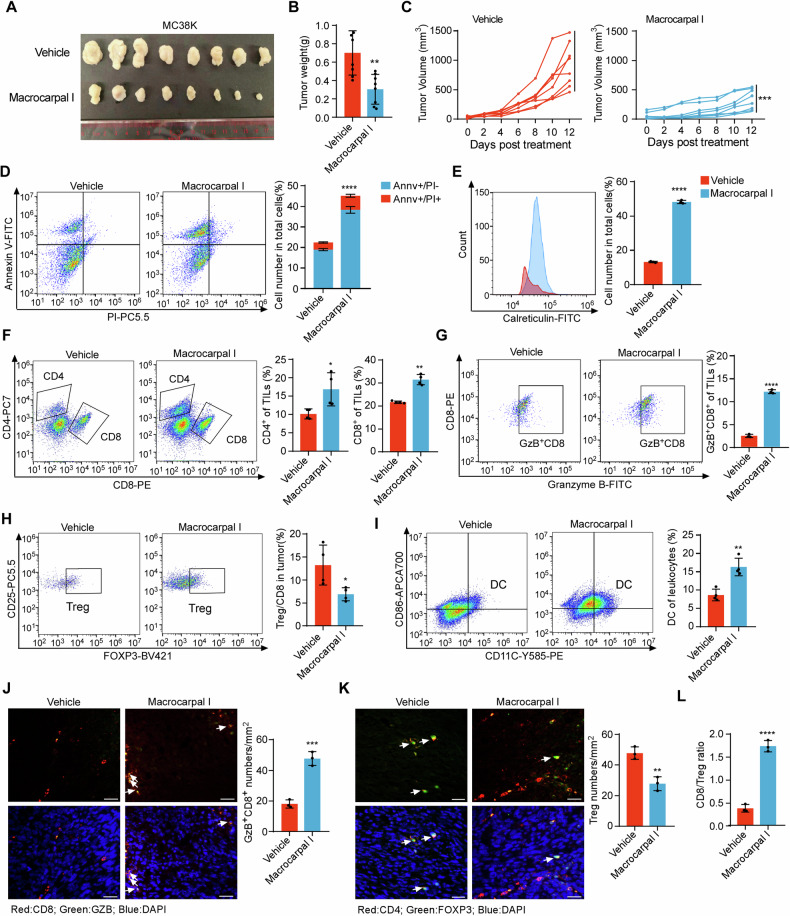
Macrocarpal I promotes anti-tumor immune response in CRC. **A** C57BL/6 J mice were subcutaneously implanted with MC38K cells and treated with either vehicle or Macrocarpal I. Tumors were collected when the experiment reached its endpoint. Each group consisted of 8 mice. **B** The weight of MC38K tumors in C57BL/6 J mice was measured and analyzed at the experimental endpoint. **C** Tumor growth curves were measured three times a week and quantified using the formula 0.5 × length × width^^2^. **D** Flow cytometry analysis of tumor cells stained with Annexin V and PI after treatment with Macrocarpal I. The dot plots and quantification data represent Annexin V^+^/PI^-^ (early-stage apoptosis) and Annexin V^+^/PI^+^ (late-stage apoptosis) cells. **E** The expression levels of cell surface calreticulin, in response to vehicle or Macrocarpal I treatment, were measured using flow cytometry on tumor cells. **F**–**I** Flow cytometry analysis of CD8^+^ T cells, Granzyme B^+^ CD8^+^ T cells, CD4^+^ T cells, FOXP3^+^ Tregs and DC infiltrated in MC38K tumors (C57BL/6 J mice). **J** Representative images of Granzyme B^+^ CD8^+^ T cells analyzed by IF staining in MC38K tumors. Scale bar, 50 μm. The right bars depict the quantification and statistical analysis of Granzyme B^+^ CD8^+^ T cells. **K** Representative images of FOXP3^+^ Tregs analyzed by IF staining in MC38K tumors. Scale bar, 50 μm. The right bars depict the quantification and statistical analysis of Tregs. **L** The ratio of CD8^+^ T cells to FOXP3^+^ Tregs was calculated based on the data from Fig. 5G, I. In B, C, D, E, F, G, H, I, J, K, L, mean ± SD, two-tailed *t*-test, ns, no significance, **p < 0.01, ***p < 0.001, ****p < 0.0001.

Given the ability of Macrocarpal I to promote ICD in CRC cells, we examined its effects on anti-tumor immune infiltration using the MC38K syngeneic mouse models [22]. Macrocarpal I induced significant ICD phenotypes in MC38K cells (Fig. S5A–C). By measuring tumor weight and volume, mice bearing MC38K tumors were highly sensitive to Macrocarpal I (Fig. 5A–C) without obvious side effects (Fig. S5D). Macrocarpal I treatment markedly reduced levels of β-tubulin and activity of PARP1 compared with vehicle (Fig. S5E, F). Flow cytometry analysis of tumor epithelial cells showed a significant increase in apoptotic cells after Macrocarpal I treatment (Fig. 5D), with a remarkable enhancement of ICD activation (Fig. 5E). Moreover, Macrocarpal I significantly increased the infiltration of total T cells (Fig. S5G), CD4^+^ and CD8^+^ T cells (Fig. 5F), Granzyme B^+^ CD8^+^ T cells (Fig. 5G, J) and dendritic cells (Fig. 5I). However, Macrocarpal I treatment dramatically decreased the infiltration of FOXP3^+^ CD4^+^ regulatory T cells (Fig. 5H, K). Furthermore, the CD8^+^/Treg ratio was significantly higher in the group treated with Macrocarpal I compared to vehicle groups (Fig. 5L). These data suggest that Macrocarpal I is a promising anti-tumor drug and can induce an immune-stimulatory tumor microenvironment.

### Macrocarpal I conquers immunotherapy resistance of anti-PD-1 in CRC

**Fig. 6:**
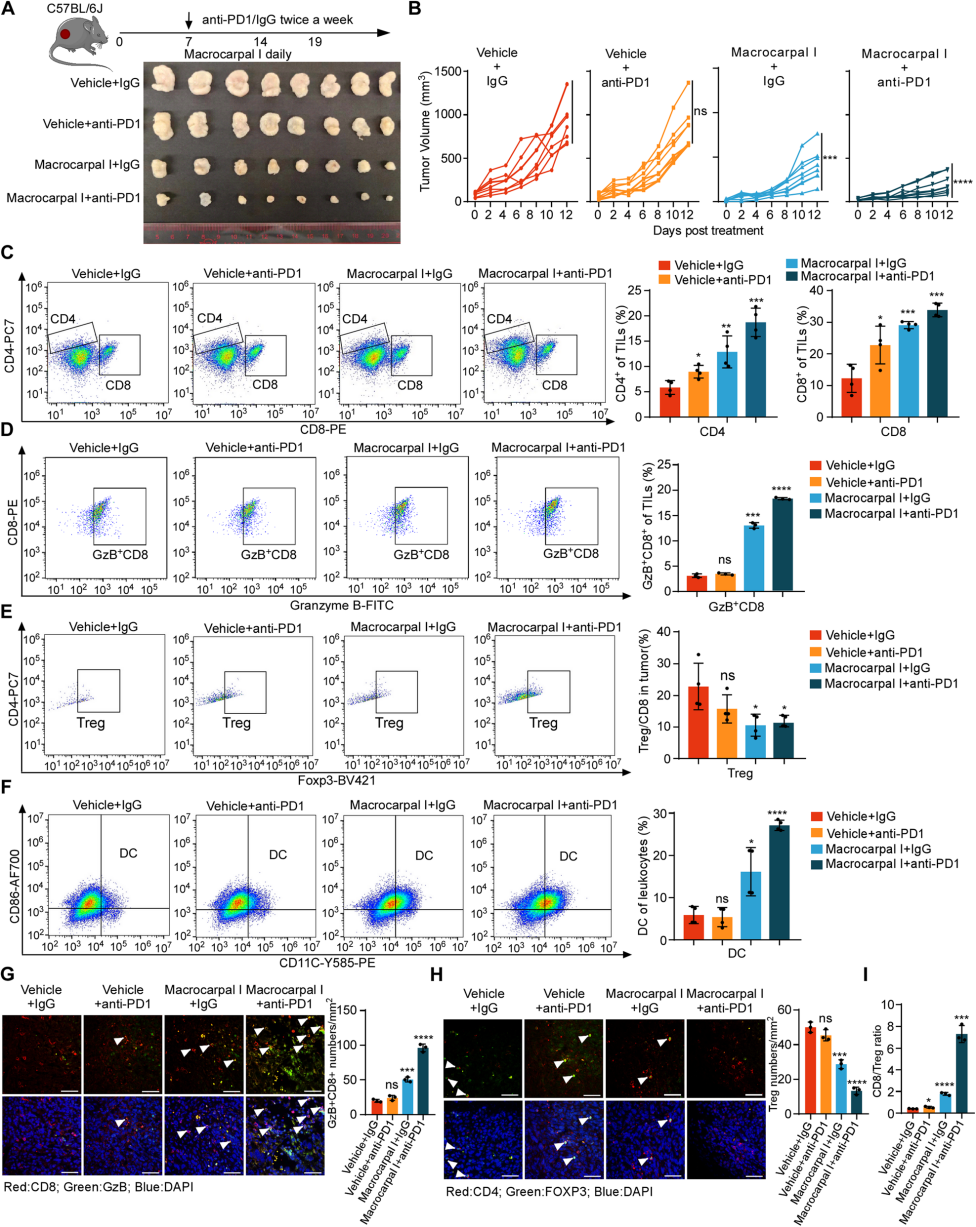
Macrocarpal I conquers immunotherapy resistance of anti-PD-1 in CRC. **A**, **B** C57BL/6 J mice were subcutaneously implanted with MC38K cells and treated with isotype IgG plus DMSO (vehicle control), anti-PD-1, Macrocarpal I or a combination of anti-PD-1 and Macrocarpal I. At the experimental endpoint, the mice were sacrificed and tumor samples were collected (A). Tumor growth was monitored and recorded over time (B). Eight mice were in each group. **C**–**F** Flow cytometry analysis was conducted to assess the infiltration of CD8^+^ T cells, CD4^+^ T cells, Granzyme B^+^ CD8^+^ T cells, FOXP3^+^ Tregs and DC in MC38K tumors of C57BL/6 J mice. IF staining was used to analyze and quantify Granzyme B^+^ CD8^+^ T cells (**G**) and FOXP3^+^ Tregs (**H**) in MC38K tumors. Representative images are shown with scale bars indicating 50 µm. The accompanying graphs present quantification and statistical analysis of Granzyme B^+^ CD8^+^ T cells and Tregs. **I** The ratio of CD8^+^ T cells to FOXP3^+^ Tregs was calculated based on the data from Fig. 6E, F. In B, C, D, E, F, G, H, I, mean ± SD, two-tailed t-test, ns, no significance, *p < 0.05, **p < 0.01, ***p < 0.001, ****p < 0.0001.

Considering the potential of Macrocarpal I to stimulate antitumor T cell infiltration, we aimed to explore the synergistic effects of combining PD-1 blockade with Macrocarpal I. While PD-1 blockade alone had limited impact on controlling MC38K tumors, the combined treatment of anti-PD-1 with Macrocarpal I led to significant tumor growth reductions (Fig. 6A, B; Fig. S6A) without noticeable side effects (Fig. S6B). ELISA and immunofluorescence confirmed that the activity of PARP1 and the expression of β-tubulin were significantly reduced after Macrocarpal I treatment or combination therapy (Fig. S6C–E). Flow cytometry analysis of tumor epithelial cells showed a significant increase in apoptotic cells after monotherapy with Macrocarpal I or combined with anti-PD1 (Fig. S6F), with a marked enhancement of the ICD effect (Fig. S6G). Flow cytometry analysis demonstrated a notable increase in the infiltration of total T cells (Fig. S6H), CD4^+^ T cells CD8^+^ T cells and Granzyme B^+^ CD8^+^ T cells (Fig. 6C, D) with a decrease in Tregs under the combination therapy (Fig. 6E). Simultaneously, there was a substantial increase in intratumoral DCs cell population with the combination treatment (Fig. 6F). Additionally, Macrocarpal I enhanced the infiltration of cytotoxic CD8^+^ T cells (Fig. 6G) while suppressing Treg infiltration seen with anti-PD-1 monotherapy (Fig. 6H). The CD8^+^/Treg ratio was markedly higher in the combination group compared to the single-agent groups (Fig. 6I). These results suggested that Macrocarpal I sensitizes CRC to ICIs.

## Discussion

In this study, we have identified Macrocarpal I as a novel dual-targeted immunomodulatory agent inducing ICD, apoptosis and ferroptosis. Mechanistically, Macrocarpal I directly targets TUBB2B and PARP1, disrupting microtubule polymerization and DNA repair processes. Importantly, immunotherapy with Macrocarpal I enhanced the anti-tumor efficacy and sensitized tumors to anti-PD-1 therapy in the MC38 syngeneic mouse model.

Natural compounds have garnered considerable attention in cancer therapy owing to their low toxicity, cost-effectiveness, and ability of impeding cancer cell growth by disrupting various critical mechanisms involved in cancer progression. The discovery of natural compounds with potent ICD-inducing capabilities holds prospects in cancer immunotherapy agents. So far, only a limited number of natural compounds trigger ICD, including doxorubicin or mitoxantrone [23]. These naturally derived compounds are believed to act as immunoadjuvants by eliciting cancer stress responses and the following release of damage-associated molecular patterns (DAMPs), including CRT, ATP and HMGB1. They bind to scavenger receptors and CD91, P2X purinoceptor 7 (P2RX7), and Toll-like receptor 2/4 (TLR2/4) on immature DCs respectively, facilitating the attraction and maturation of DCs capable of priming or reactivating tumor-specific T cells [24]. Macrocarpal I is a natural compound that possesses various biological activities, including antioxidant, antimicrobial, and anticancer effects [14, 15]. In the current study, the increased level of surface-exposed CRT, secreted-ATP, and extracellular released-HMGB1 were observed in CRC cells following Macrocarpal I treatment, indicating the potential of Macrocarpal I as a novel ICD inducer. Moreover, Macrocarpal I activated the PERK/eIF2a/ATF4/CHOP signaling pathway which exerts the pro-apoptosis effect as a typical downstream of ER stress [25]. The apoptotic phenotype observed in vivo and in vitro under Macrocarpal I treatment further experimentally validated that Macrocarpal I promotes cancer cell apoptosis through activating the PERK/eIF2a/ATF4/CHOP signaling pathway. Based on these findings, we propose that the natural compound Macrocarpal I may serve as a novel potential anti-tumor drug and immunotherapy adjuvant.

Through mass spectrometry and docking analysis, we elucidate that Macrocarpal I is a potential drug to disrupt microtubule polymerization by targeting β-tubulin (TUBB2B). Microtubule-associated processes, including polymerization and depolymerization, function heavily in regulating cell division, organelle positioning, and transport. Data in this study support that Macrocarpal I induces G2/M phase arrest by disrupting microtubule polymerization, which is concordant with established tubulin inhibitors [26]. Microtubule-targeting agents (MTAs) are turbulent in the microtubule dynamics by binding to the microtubules via different mechanisms. Docetaxel and paclitaxel are proposed as well-known compounds for chemotherapy in prostate tumors and triple-negative breast cancer, they share functional characteristics of pharmacologic perturbations of microtubule depolymerization, inducing cell cycle arrest [27]. Recent studies have revealed various genes that contribute to the modulation of microtubule stability, resulting in resistance to paclitaxel [28, 29]. In contrast, Macrocarpal I belongs to a class of non-taxane alkaloid microtubule inhibitors. Macrocarpal I specifically binds to the colchicine-binding site of tubulin, consequently inhibiting its polymerization into microtubules. Colchicine-binding site inhibitors have several advantages over colchicine and other MTAs, including their ability to overcome ABC-transporter-mediated multidrug resistance and the overexpression of β-tubulin [30], and their vascular disrupting activity [31]. Thus, the mechanism of action of Macrocarpal I on tubulin differs from that of paclitaxel, suggesting that they may act synergistically, and combination therapy may reverse paclitaxel resistance. Therefore, the application of Macrocarpal I may broaden the population that benefits from paclitaxel therapy.

Microtubules play a crucial role in protein transport mediated by microtubule-associated proteins (MAPs) and molecular motor proteins. MAPs such as kinesin and dynein facilitate the transportation of vesicles and proteins from the ER membrane to other cellular regions through their interaction with microtubules [32]. The three ER membrane proteins, CLIMP63, p180, and KTN1, bind to microtubules to maintain ER morphology [33]. The dynamic process of microtubule polymerization and depolymerization is vital for ER homeostasis, the destabilization of which leads to ER stress and ICD response. The data above suggest that the interference of microtubule polymerization by Macrocarpal I could probably induce ER stress, activating ICD-related apoptosis.

In addition, PARP1 was identified as another target of Macrocarpal I. We confirmed the potential of Macrocarpal I to stimulate H2A.X phosphorylation. H2A.X serves as a marker for double-strand DNA breaks and is indicative of PARP1 inhibition [20]. And the DNA mismatch repair signaling pathway was enriched under Macrocarpal I treatment as determined by RNA-seq analysis in our previous work [15]. Recent studies have suggested that PARP enzymes are crucial regulators of DNA damage response (DDR) and are involved in the development, progression, and therapeutic response of cancers [34]. The most notable example so far is the use of PARP inhibitors (PARPi) to treat individuals with inherited breast and ovarian cancers lacking wild-type copies of the BRCA1 and BRCA2 genes [28]. BRCA1 and BRCA2 are two critical tumor suppressor genes crucial for DNA double-strand break repair [29]. However, the limited activity and indications, as well as drug resistance of PARPi, have been revealed [35]. Notably, PARPi has not been approved for treating gastrointestinal cancers. In a previously conducted mutational signature analysis, only 7% to 12% of patients with gastric carcinoma harbored intrinsic DNA repair defects [36]. Emerging evidence suggests that MTAs may potentiate PARPi-induced DNA damage by impairing the trafficking of proteins critical for double-strand DNA damage repair [37]. By synergizing with PARPi, which inhibits the alternative DNA repair pathway, MTAs prolong DNA damage and enhance cytotoxicity. Therefore, Macrocarpal I serves as an immune adjuvant and simultaneously exerts a PARPi effect, it is expected to overcome the challenge of immunotherapy resistance in cancer.

In summary, this study identifies a novel dual-targeted immunomodulatory agent with potent anti-tumor effects. Mechanistically, Macrocarpal I directly targets TUBB2B and PARP1, exerting candidate merits for overcoming resistance to paclitaxel or PARP inhibitors. However, a limitation of Macrocarpal I is its notably high IC50 value. Our ongoing research aims to optimize the molecular structure of Macrocarpal I through chemical modification or formulation approaches, to improve its bioavailability. Additionally, pharmacokinetic studies are required to investigate the absorption, distribution, metabolism, and excretion (ADME) properties of Macrocarpal I, further determining its clinical feasibility.

## Materials and methods

### Tumor models and treatments

Animal experiments in this study were approved by the Experimental Animal Ethics Committee, Sun Yat-sen University Cancer Center (approval number #L025504202108006). The animals were maintained in pathogenfree conditions and cared for in accordance with the policies and accreditation of the Council for the International Organizations of Medical Sciences.

Female C57BL/6 J mice, aged 6–8 weeks, were procured from the Sun Yat-Sen University Cancer Center (Guangzhou, China) and housed in a controlled environment with a 12 h light/dark cycle at 18–22 °C and 50–60% humidity. All experimental procedures were approved by the Institutional Animal Care and Use Committee of Sun Yat-sen University. Mice were randomly grouped (*n* = 8/group) and received a subcutaneous injection of MC38K cells (1 × 10^5^ cells/mouse) on day 0. After tumor growth to about 5 mm in diameter, mice received intraperitoneal injections of anti-PD-1 monoclonal antibody (BE0146, clone RMP1-14; Bio-XCell) and IgG isotype control (BE0089, clone 2A3; Bio-XCell) at a dosage of 200 μg/injection, administered once every two days. Macrocarpal I (Shanghai Acmec Biochemical Co, Ltd, Shanghai, China) was administered once daily via intraperitoneal injections at a dosage of 60 mg/kg, starting from tumor inoculation and continuing for 7 days. Tumor measurements were conducted thrice weekly. At the endpoint, mice were euthanized while the tumor diameter did not exceed 20 mm, and the tumors were excised, fixed in formalin, and subsequently embedded in paraffin.

### Cell lines, culture conditions and viral infection

The MC38 cell line, a C57BL/6 J mouse colon adenocarcinoma cell line, was generously provided by Dr. Xiaojun Xia from the State Key Laboratory of Oncology in South China, Sun Yat-Sen University Cancer Center, Guangzhou, China. The cell line was originally obtained from Kerafast (Boston, MA, USA). Human colorectal cancer cell lines DLD1 and SW620 were obtained from ATCC (Manassas, VA, USA). Cells were cultured in RPMI 1640 medium (Thermo Scientific, Waltham, MA, USA) supplemented with 10% fetal bovine serum (FBS) (Gibco, Grand Island, NY, USA). Lentiviral transduction was performed by transfecting 293 T cells with pLenti6.3 plasmids encoding KRAS^G12D^ using Lipofectamine 2000 Transfection Reagent (11668019, Thermo Fisher). After 24 to 48 h, the supernatants were filtered through a 0.45 μm nylon filter and then added to the cultured cancer cells. After three days, cells were subjected to selection with blasticidin (3 μg/ml) for one week. Western blotting analysis was performed to confirm the transduction efficiency. We knockdown the human TUBB2B gene with the Lenti-CrisprV2 plasmid (sg#1: CTAACCGAATCCATCGTGCC, sg#2: CAGGACCGAGTCGACCAGCT), and knockdown the human PARP1 gene (sg#1: CGATGCCTATTACTGCACTG, sg#2: CTTTATCCTCTGTAGCAAG G).

### Single-cell isolation and flow cytometry assay

Single-cell isolation and flow cytometry assays were performed as previously described [22]. Briefly, CRC tumor single cells were isolated using the Mouse Tumor Dissociation Kit (130-096-730, Miltenyi Biotec) according to the standard protocol. Digested tumors were filtered through 70 μM filters into RPMI-1640 and centrifuged at 300 × *g* for 5 min at 4 °C. Erythrocytes were depleted from all single cells using red cell lysis buffer for 10 min at room temperature. A maximum of 2 × 10^6^ cells per tumor were blocked with Blocking Reagent (130-059-901, Miltenyl Biotec) for 10 min at room temperature. The single-cell samples were stained with Ghost Dye Violet 450 in the dark for 15 min and then with the indicated antibodies on ice for 30 min. Antibodies for DC included CD45 (APC-Cy7, clone 30-F11, 103116, BioLegend), CD11b (BV605, clone M1/70, 101237, BioLegend), CD11c (PE, clone N418, 117308, BioLegend), and CD86 (AF700, clone GL-1, 105024, BioLegend). Antibodies for T cells included CD45 (APC-Cy7, clone 30-F11, 103116, BioLegend), CD3e (APC, clone 145-2C11, 100312, BioLegend), CD4 (PE-Cy7, clone GK15, 100422, BioLegend), CD8a (PE, clone S18018E, 100707, BioLegend), Granzyme B (FITC, clone QA18A28, 396404, BioLegend), CD25 (PerCP/Cy5.5, clone 3C7, 101911, BioLegend), and FOXP3 (BV421, clone MF14, 126419, BioLegend). Flow cytometry analysis was performed using a flow cytometer with CytExpert software, and the percentages of each cell population were analyzed using FlowJo software.

### Flow cytometry analyses

Calreticulin was determined by flow cytometry. Cells (1 × 10^6^ cells/ml) were treated with Macrocarpal I, then harvested, washed with cold PBS, and stained with anti-calreticulin rabbit (bs-5913R, Bioss). Subsequently, cells were rinsed with cold PBS and incubated with Alexa Fluor®488-conjugated secondary antibodies (Abbkine, China) for 30 min at room temperature in the dark. Finally, cells were subjected to flow cytometry. A minimum of 10,000 cells were acquired per sample, and histograms were analyzed using FlowJo software.

### Immunofluorescence assay

Paraffin-embedded samples were cut into 4 mm sections. Antigen retrieval was carried out in a citrate solution using a microwave oven (95 °C, 30 min). For cell samples, 5 × 10^4^ cells were plated on glass coverslips overnight and exposed to specified drugs for a set duration. Following treatment, cells were fixed with fresh 4% formaldehyde (methanol-free) for 15–20 min. Slides or coverslips were then blocked with 2% bovine serum albumin in PBS for 1 h at room temperature. Primary antibodies included anti-CD8 rat (ab22378, Abcam), anti-Granzyme B mouse (MA1-80734, Invitrogen), anti-CD4 rabbit (ab183685, Abcam), anti-FOXP3 mouse (14-4777-82, Invitrogen), anti-calreticulin rabbit (bs-5913R, Bioss), anti-TUBB2B rabbit (K113526P, Solarbio), anti-α-tubulin mouse (RM2007, Beijing Ray), anti-β-tubulin rabbit (AF7011, Affinity), anti-phospho-H2A.X (Ser139) rabbit antibody (AF5836, Beyotime), and anti-phospho-PERK rabbit antibody (AP1420, ABclonal). Subsequently, slides or coverslips were rinsed with cold PBS and incubated with Alexa Fluor®488, Alexa Fluor®594-conjugated secondary antibodies (Abbkine, China), phalloidin-594-conjugated (CA1680, Solarbio) or ER tracker (C1041S, Beyotime) for 30 min at room temperature in the dark. Finally, staining with DAPI was done, and the images were examined under a microscope.

### Cell cycle phase distribution

Cell cycle analysis by flow cytometry was carried out as previously described [38]. In brief, 1 × 10^6^ cells/ml were seeded in 6-well plates and cultured in 10% 1640 medium for 24 h. After synchronization, the cells were treated with Macrocarpal I and processed as previously stated [39]. Following fixation, cells were resuspended in PBS, stained with propidium iodide (421301, Biolegend), and subjected to flow cytometry. A minimum of 10,000 cells were acquired per sample, and histograms were analyzed using FlowJo software.

### Annexin V-FITC/PI double-staining assay

During apoptosis, the integrity of the cell membrane is disrupted and phosphatidylserine is exposed. Annexin V conjugated with FITC (KGF001, KeyGEN) selectively binds to phosphatidylserine, allowing for the detection of early and late apoptotic cells. Cells (1 × 10^6^ cells/ml) were treated with Macrocarpal I, then harvested, washed with cold PBS, and subjected to Annexin-PI double-staining assay [40]. Samples were resuspended in 1 × binding buffer and incubated with Annexin V-FITC (0.4 g/μl) and PI (0.05 mg/ml), followed by analysis via flow cytometry. A minimum of 10,000 cells were acquired for each sample and illustrated as a dot plot using FlowJo software.

### Hoechst 33342 staining assay

Nuclear morphology was detected using the method of Araki et al. [41]. Macrocarpal I-treated cells were fixed with methanol acetic acid for 10 min, followed by staining with Hoechst 33342 (C1029, Beyotime) at room temperature in the dark for 5 min. The cells were then washed twice with PBS, examined, and immediately photographed under an Olympus inverted fluorescence microscope with an excitation wavelength of 330–380 nm. Apoptotic cells were identified based on nuclear morphology changes, such as chromatin condensation and fragmentation.

### Senescence-associated β-galactosidase staining

The senescence of the cell was identified by a β-galactosidase (SA-β-gal) staining kit (C0602, Beyotime Biotechnology). In brief, cells were fixed in β-galactosidase staining fixative for 15 min at room temperature. After washing with PBS buffer, the cells were incubated with the staining working solution overnight at 37 °C without CO2. Part of the stained cells was added to a glass slide or a six-well plate using citric acid buffer and observed under a normal optical microscope.

### HMGB1-Gaussia luciferase assay

Cells were seeded at a density of 1 × 10^5^ cells/well in 24-well plates one day prior to transfection. Lipofectamine 2000 reagent (Invitrogen, USA) was used to transfect the cells with the HMGB1 gaussia luciferase reporter plasmid. After 48 h, gaussia luciferase activities were measured using the Gaussia Luciferase Assay Kit (ab189814, Abcam) according to the manufacturer’s instructions. The results were normalized to Gaussia luciferase values, and all experiments were conducted at least three times with data presented as mean ± SD.

### Extracellular ATP assay

Extracellular ATP in the conditioned medium following the indicated treatment was measured using a luciferin-based Enhanced ATP Assay Kit (S0027, Beyotime Biotechnology) and according to the manufacturer’s instructions. Luminescence was measured using BioTek Synergy H1, and the luminescence (RLU) of ATP standards was plotted against their corresponding concentrations. The concentrations of extracellular ATP in the conditioned medium were determined directly from the standard curve. All experiments were conducted at least three times, and the data are presented as the mean ± standard deviation (mean ± SD).

### Drug affinity responsive target stability (DARTS)

Lysates from SW620 and DLD1 cells were incubated with 10 and 100 μM Macrocarpal I or without the compound for 9 h at room temperature [42]. Lysates were then divided into seven parts and digested with different concentrations of Pronase E (1074330001, Sigma Aldrich) for 30 min at room temperature. The digestion was stopped by boiling the sample immediately after adding the loading buffer. Western blot was performed using a 12% SDS-PAGE gel for each sample.

### PARP1 enzyme assay

PARP1 activity was measured by the PARPtrap™ Assay Kit for PARP1 (17-10149, Merck Millipore) according to the guidelines specified in the manufacturer’s instructions. To perform the assay, PARP enzyme, β-NAD, activated DNA, test compounds, and recombinant nicotinamide enzyme were combined and incubated for 30 min. During the incubation, the activated DNA triggers PARP1 to produce poly(ADP-ribose) and nicotinamide. In a secondary reaction, the nicotinamide enzyme converts nicotinamide into niacin and NH3+ (free ammonia). To generate a readout signal, a proprietary developer reagent is added, and the signal is read using a fluorescence plate reader.

### Western blot analysis

Total cell lysates were extracted using RIPA buffer (89900; Pierce Biotechnology) and the protein concentration was determined using a bicinchoninic acid (BCA) assay according to the manufacturer’s instructions. Western blotting analyses were performed using the following antibodies: PERK rabbit antibody (A21255, ABclonal), phospho-PERK rabbit antibody (AP1420, ABclonal), eIF2A rabbit antibody (A9709, ABclonal), phospho-eIF2A rabbit antibody (AP0692, ABclonal), ATF4 rabbit antibody (A0201, ABclonal), DDIT3/CHOP rabbit antibody (A11346, ABclonal), TUBB2B rabbit antibody (K113526P, Solarbio), α-tubulin mouse antibody (RM2007, Beijing Ray), β-tubulin rabbit antibody (AF7011, Affinity), β-actin rabbit antibody (AF7018, Affinity), phospho-H2A.X (Ser139) rabbit antibody (AF5836, Beyotime), PARP1 rabbit antibody (AG1040, Beyotime), and GAPDH rabbit antibody (AF7021, Affinity). After secondary incubation with goat anti-rabbit IgG (RM30021L, Beijing Ray) and goat anti-mouse IgG (RM3001L, Beijing Ray), ECL solution was used to visualize the bands. The signals were recorded using a chemiluminescence imaging analysis system.

### Cellular microtubule stabilization assay

Following treatment with Macrocarpal I, DLD1 and SW620 cells were harvested in lysis buffer (100 mM PIPES pH 6.9, 1 mM EGTA, 1 mM MgCl2, 30% glycerol, 5% DMSO, 1% NP-40, 5 mM GTP, and protease inhibitors). The cell lysates were then centrifuged at 180,000 × *g* at 37 °C for 1 h. The resulting supernatants containing soluble tubulin were separated from the pellets containing polymerized tubulin. Both fractions were adjusted to the same volume using SDS-PAGE loading buffer. Subsequently, the amount of β-tubulin in equal aliquots of the polymerized tubulin fraction and free tubulin fraction was determined by western blotting.

### Autodocking Vina

Autodocking vina predicts receptor-ligand interactions by using three-dimensional structures of both the ligand and the receptor in a compatible file format (PDB or PDBQT), which can be obtained from PDB (https://www.rcsb.org/) or our previous work for Macrocarpal I. The receptor structure should include information about the active site or binding pocket. Once input files and parameters are set, Autodocking vina systematically explores different orientations and conformations of the ligand within the receptor’s binding site, evaluating their binding affinity using a scoring function. After docking calculations are complete, Autodocking Vina provides a ranked list of predicted binding poses based on estimated binding affinity.

### Protein expression and purification

The human TUBB2B and PARP1 proteins were cloned into pET28a (Novagen) with an N-terminal His-tag and expressed in Escherichia coli strain BL21 (DE3) RIL. Expression was induced at 16 °C overnight with 0.2 mM isopropyl β-D-thiogalactoside (IPTG). The protein was then purified by Ni-NTA (Qiagen) affinity chromatography, loaded onto a heparin column (GE Healthcare), and eluted with a salt gradient (0-1 M NaCl). Further purification was performed using Superdex 75 size-exclusion chromatography. Purified samples were analyzed by SDS-PAGE and detected by Coomassie brilliant blue (CBB) staining.

### Isothermal titration calorimetry (ITC)

All binding experiments were performed at 25 °C on a Microcal PEAQ-ITC instrument (Malvern). In an ITC experiment, ΔH of binding is measured directly. The microcalorimeter has two cells: one contains water and serves as a reference cell, while the other contains the sample, into which a binding partner is titrated using an injection syringe. Heat sensing devices detect temperature differences between the cells when binding occurs in the sample cell and give feedback to the heaters, which compensates for this difference and returns the cells to equal temperature. Titrations were performed using standard protocols, and data were processed using the Origin 7.0 program.

### Mass spectrometry (MS) analysis

To identify the binding proteins of Macrocarpal I, DLD1 cells were treated with or without Macrocarpal I (10 and 100 μM) overnight at 4 °C. The lysates were then digested with Pronase E (1074330001, Sigma Aldrich) for 30 min at room temperature. Subsequently, the samples underwent mass spectrometry (MS) analysis. Information on the peptides and counts for Macrocarpal I-binding proteins analyzed by MS assays is provided in Fig. S3A.

### Statistical analysis

Statistical analysis was conducted using GraphPad Prism 7 or SPSS 21.0 (IBM Corp, Armonk, NY, USA). Experiments were performed with three biological replicates unless otherwise specified. The results were presented as the mean ± standard deviation of the mean from three independent experiments. Group comparisons were performed using either a *t*-test or one-way ANOVA. A significance level of *p* < 0.05 was applied to all analyses (*p < 0.05, **p < 0.01, ***p < 0.001, ****p < 0.0001).

## Supplementary information

supplementary figures and figure legends

Original western blot

## Data Availability

The data generated or analyzed for this study will be available by the corresponding author on reasonable request.
