# Supplementary material for: Macrocarpal I induces immunogenic cell death and synergizes with immune checkpoint inhibition by targeting tubulin and PARP1 in colorectal cancer
Source: Cell Death Discov. 2025 Feb 22;11:73. doi: 10.1038/s41420-025-02360-9 (PMC11846858; doi:10.1038/s41420-025-02360-9)

**Supplementary Figures And Figure Legends**

**Fig. S1** Cells were stained with calreticulin and subsequently analyzed using flow cytometry. Representative dot plots displayed the cells positive for calreticulin.

**Fig. S2** Macrocarpal I triggers apoptosis and ferroptosis in CRC cells. (A) Cellular senescence induced by Macrocarpal I was observed and recorded using an Olympus microscope. Scale bar, 50 μm. (B) The activity of the HMGB1-Gluc reporter in response to Macrocarpal I (50 μM) in combination with Z-VAD-FMK, Ferrostatin-1 and Necrostatin-1 in SW620 and DLD1 cells. (C) Flow cytometry analysis of calreticulin expression on the cell surface from SW620 and DLD1 cells following treatment with Macrocarpal I (50 μM) in combination with Z-VAD-FMK, Ferrostatin-1 and Necrostatin-1. (D) Chemiluminescence assay detection of ATP secretion from SW620 and DLD1 cells following treatment with Macrocarpal I (50 μM) in combination with Z-VAD-FMK and Ferrostatin-1. (E) The activity of the HMGB1-Gluc reporter in response to Macrocarpal I (50 μM) in combination with Z-VAD-FMK and Ferrostatin-1 in SW620 and DLD1 cells. (F) Flow cytometry analysis of calreticulin expression on the cell surface from SW620 and DLD1 cells. (G) Flow cytometry analysis of NCM460 and T cells stained with Annexin V and PI after treatment with different concentrations of Macrocarpal I (10, 50, and 100 μM). In A, B, C, D, E, F, G, mean ± SD, n = 3, two-tailed t-test, ns, no significance, **p* < 0.05, ***p* < 0.01, ****p* < 0.001, *****p* < 0.0001.

**Fig. S3** Identification of TUBB2B and PARP1 as targets of Macrocarpal I. (A-B) Mass spectrometry analysis depicts the protein coverage of TUBB2B and PARP1, along with the best unique peptide-spectrum matches (PSM). (C-D) 3D illustration shows the interaction mode of Colchicine and Paclitaxel with TUBB2B. The active site and amino acid residues are highlighted in purple, while hydrogen bonds are denoted in blue, ionic interactions in gold, and hydrophobic interactions in grey. (E-F) Detection of TUBB2B and PARP1 protein purification using Coomassie blue staining. (G) Activity assessment of recombinant PARP1 after treatment with different concentrations of Macrocarpal I. In G, mean ± SD, n = 3, two-tailed t-test, *****p* < 0.0001.

**Fig. S4** KnockdownTUBB2B and PARP1 induced ICD responses and cell death. (A) SW620 and DLD1 cells were treated with the indicated concentrations of drugs for 24 h, and stained with α‐tubulin was examined by IF. Scale bar, 50 μm. (B) SW620 and DLD1 stable cells were subjected to western blot assay for TUBB2B and PARP1. (C) Representative confocal microscopy images of endogenous p-PERK clusters and ER visualized by IF staining of p-PERK (green) along with the ER tracker (red) in cells knockdown TUBB2B. Scale bar, 50 μm. (D-E) The chemiluminescence assay was utilized to detect ATP secretion in cell supernatants. (F-G) The activity of the HMGB1-Gluc reporter was meticulously analyzed within SW620 and DLD1 stable cells. (H-I) The expression levels of cell surface calreticulin were measured using flow cytometry on SW620 and DLD1 stable cells. (J) Flow cytometry analysis of SW620 stable cells stained with Annexin V and PI. (K) Flow cytometry analysis of liperfluo expression levels in SW620 stable cells. In A, C, D, E, F, G, H, I, J, K, mean ± SD, n = 3, two-tailed t-test, **p* < 0.05, ***p* < 0.01, ****p* < 0.001, *****p* < 0.0001.

**Fig. S5** Macrocarpal I induces immunogenic cell death in MC38K cells. (A) Flow cytometry was used to measure cell surface calreticulin expression levels in MC38K cells following treatment with DMSO or Macrocarpal I. Three replicates were performed per group. (B-C) ATP secretion in cell supernatants was quantified using a chemiluminescence assay. Three replicates were performed per group. (D) Mean ± SD of mouse weight measurements taken three times a week and presented as weight-time curves. (E) Representative images of TUBB2B^+^ and β-tubulin^+^ cells analyzed by IF staining in MC38K tumors. Scale bar, 50 μm. (F) The activity of PARP1, in response to vehicle or Macrocarpal I treatment, was measured using ELISA. (G) Flow cytometry analysis of total T cells infiltrated in MC38K tumors (C57BL/6J mice). In A, B, C, E, F, G, mean ± SD, n = 3, two-tailed t-test, **p* < 0.05, ***p* < 0.01, ****p* < 0.001, *****p* < 0.0001.

**Fig. S6** Combined therapy of anti-PD-1 with Macrocarpal I are associated with Figure 6. (A) The weight of MC38K tumors in C57BL/6J mice at the endpoint. (B) Mouse weights were measured thrice weekly and depicted as weight-time curves. (C) The activity of PARP1 was measured using ELISA. (D-E) Representative images of TUBB2B^+^ (D) and β-tubulin^+^ (E) cells analyzed by IF staining in MC38K tumors. Scale bar, 50 μm. (F) Flow cytometry analysis of tumor cells stained with Annexin V and PI after treatment. (G) The expression levels of cell surface calreticulin were measured using flow cytometry on tumor cells. (H) Flow cytometry analysis of total T cells in MC38K tumors from C57BL/6J mice. In A, C, D, E, F, G, H, mean ± SD, two-tailed t-test, ns, no significance, **p* < 0.05, ***p* < 0.01, ****p* < 0.001, *****p* < 0.0001.


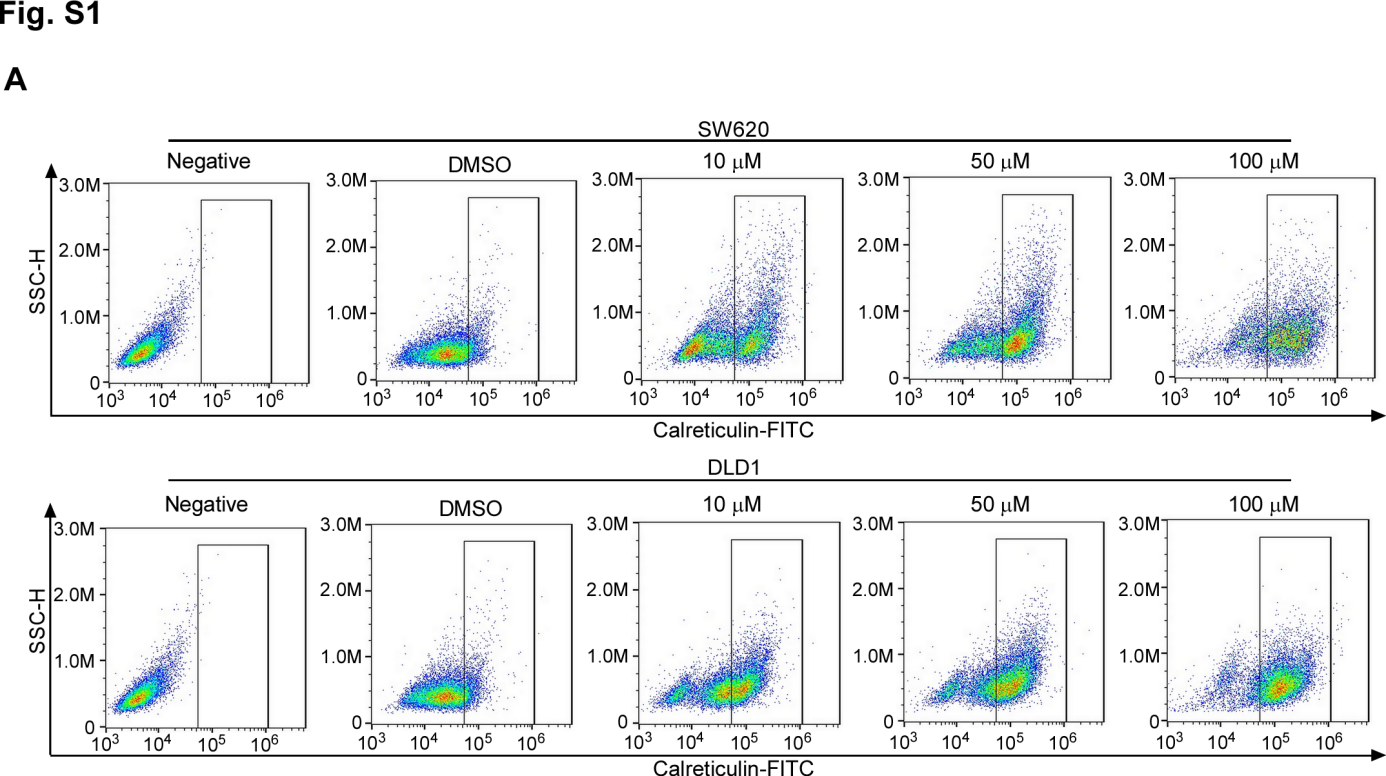


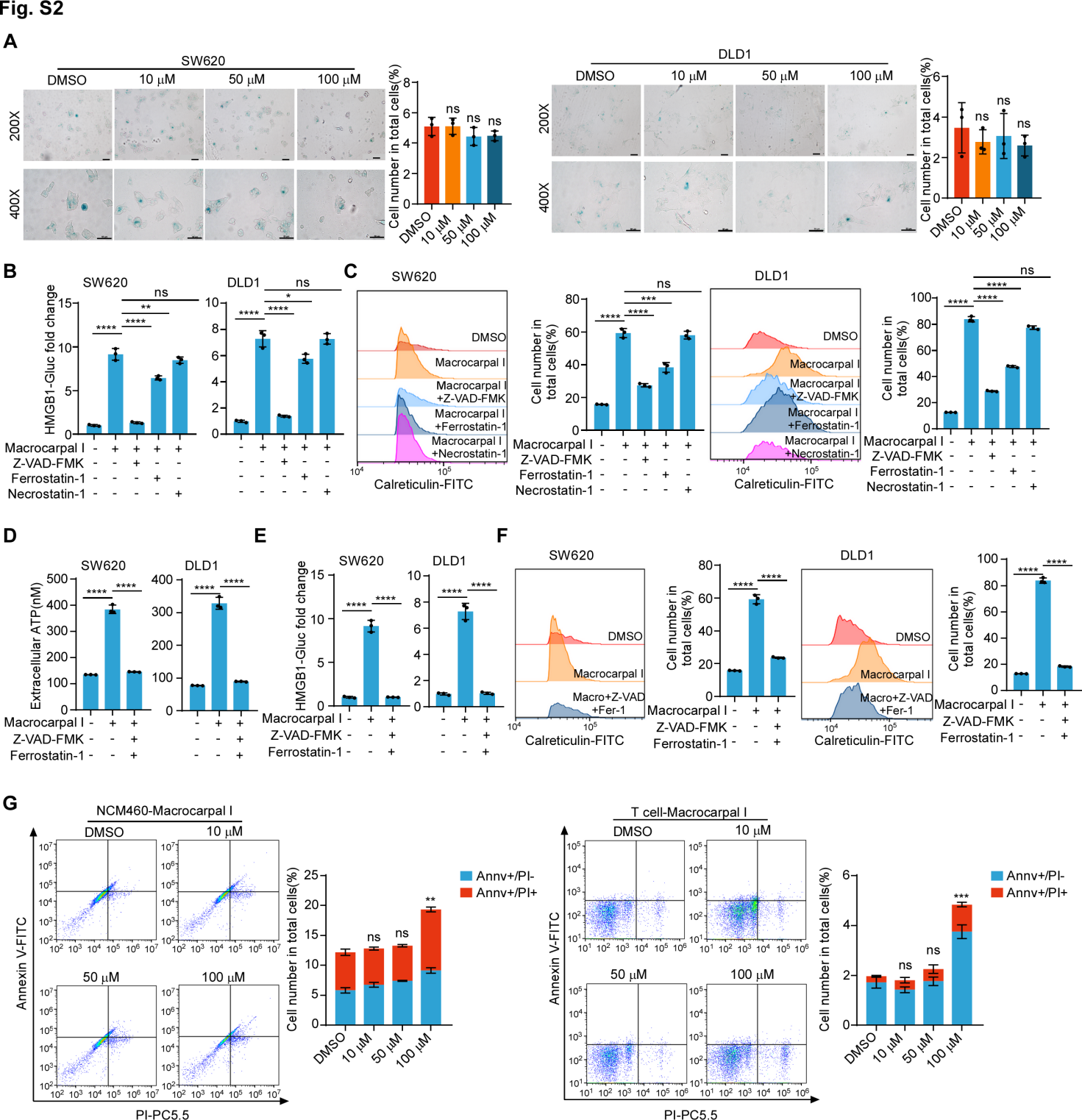


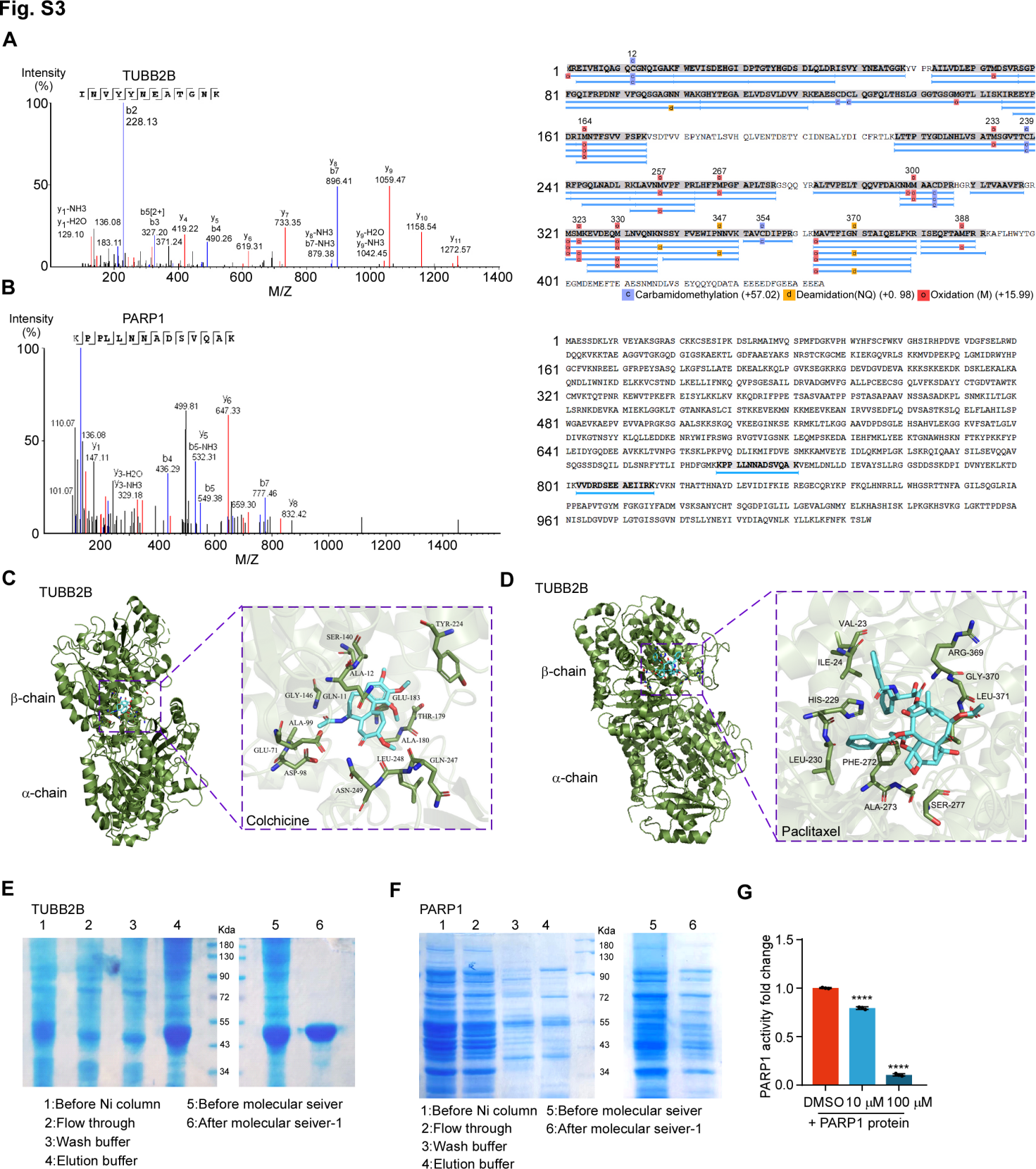


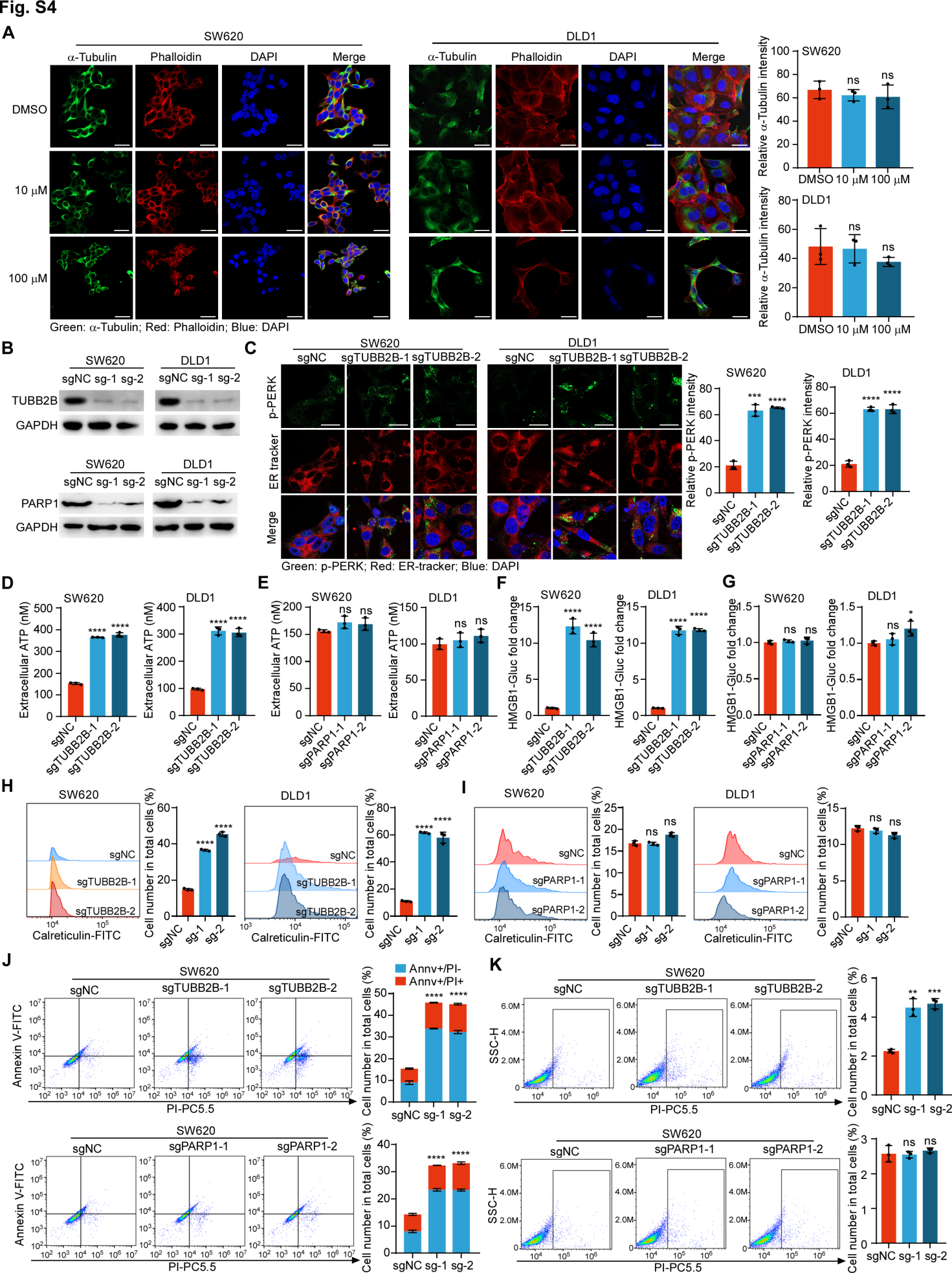


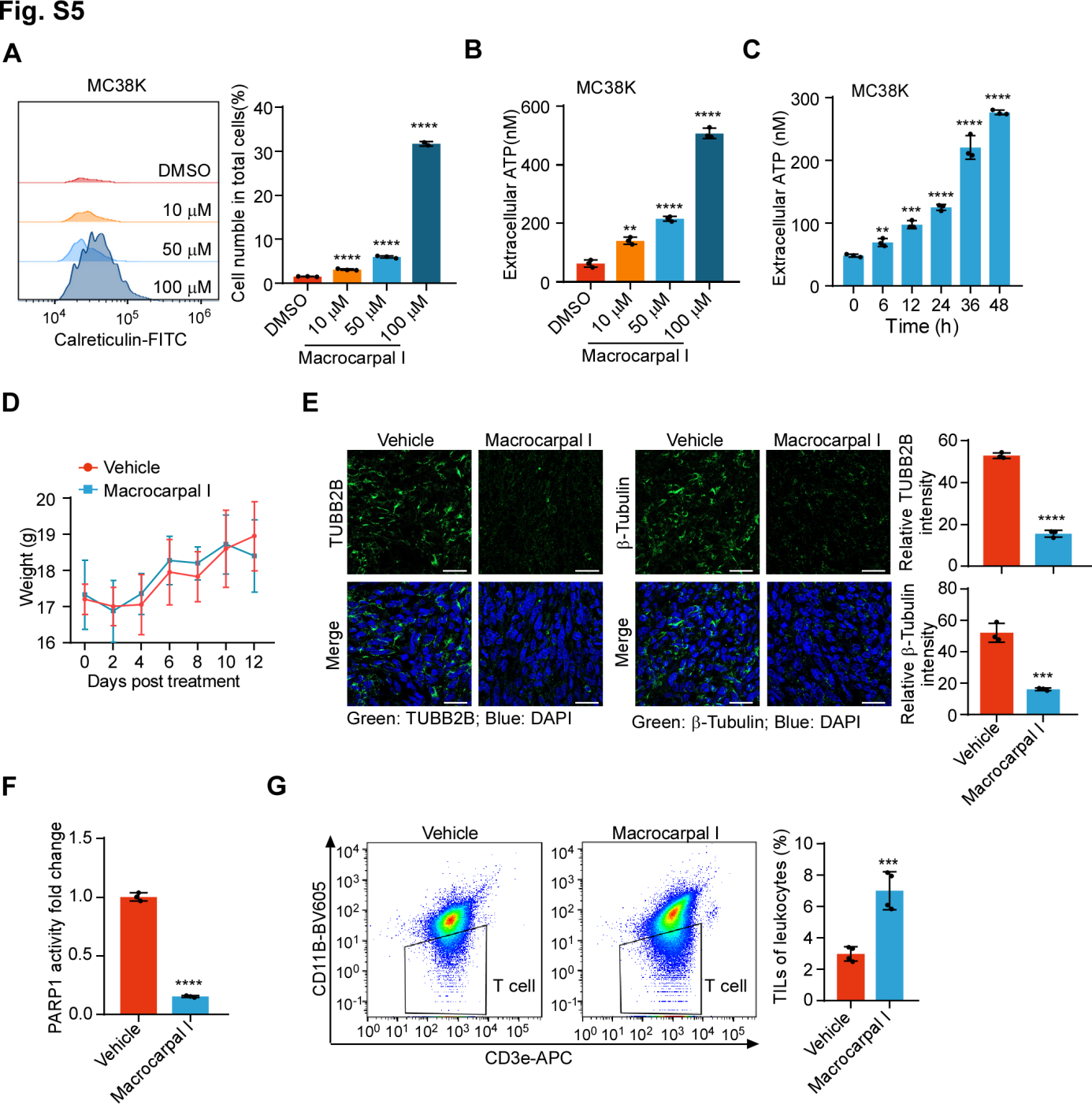


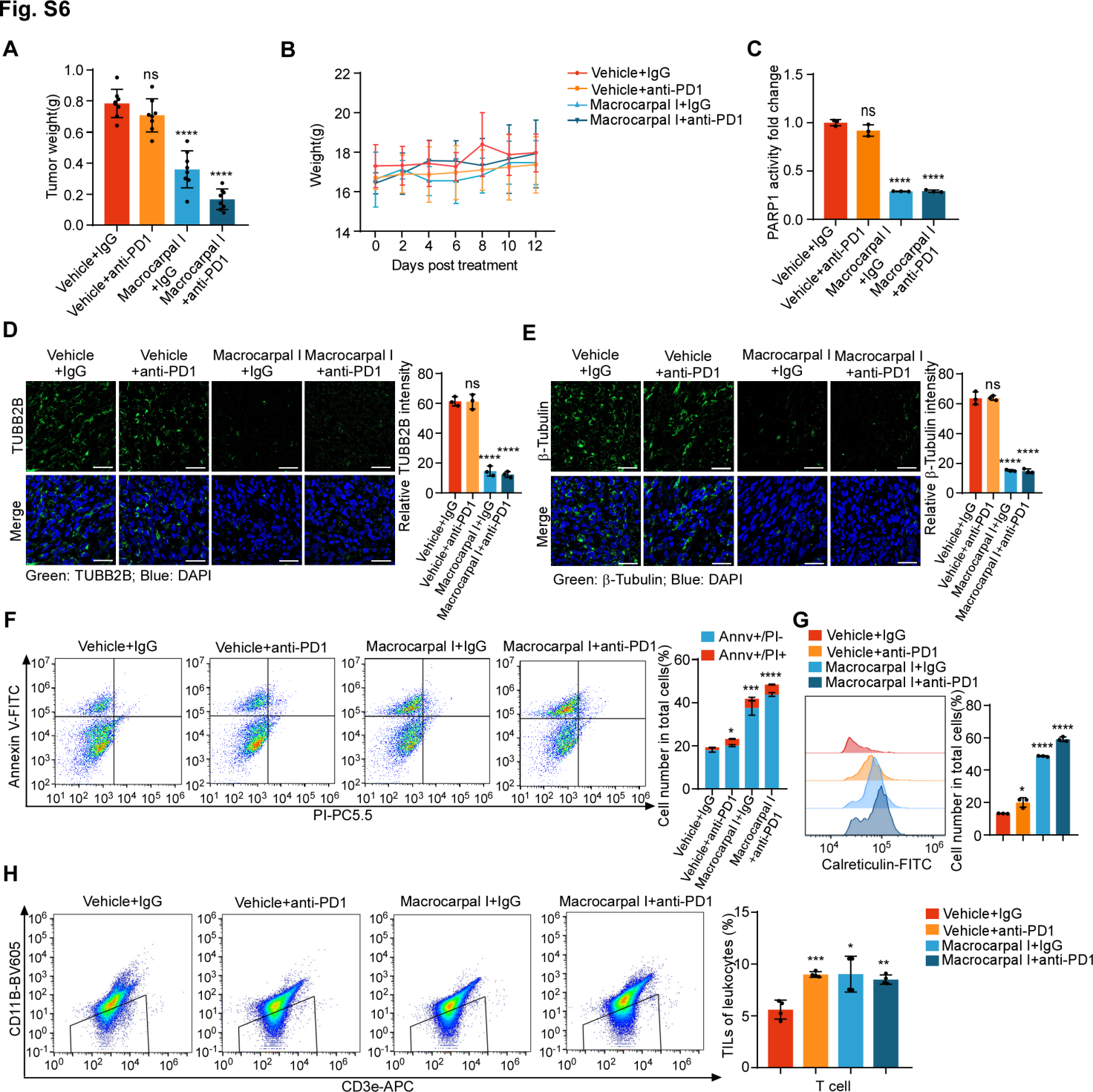

Supplement: Supplementary file 1 — supplementary figures and figure legends [file 41420_2025_2360_MOESM1_ESM.docx]
