# Supplementary figures and images for: Macrocarpal I induces immunogenic cell death and synergizes with immune checkpoint inhibition by targeting tubulin and PARP1 in colorectal cancer
Source: Cell Death Discov. 2025 Feb 22;11:73. doi: 10.1038/s41420-025-02360-9 (PMC11846858; doi:10.1038/s41420-025-02360-9)

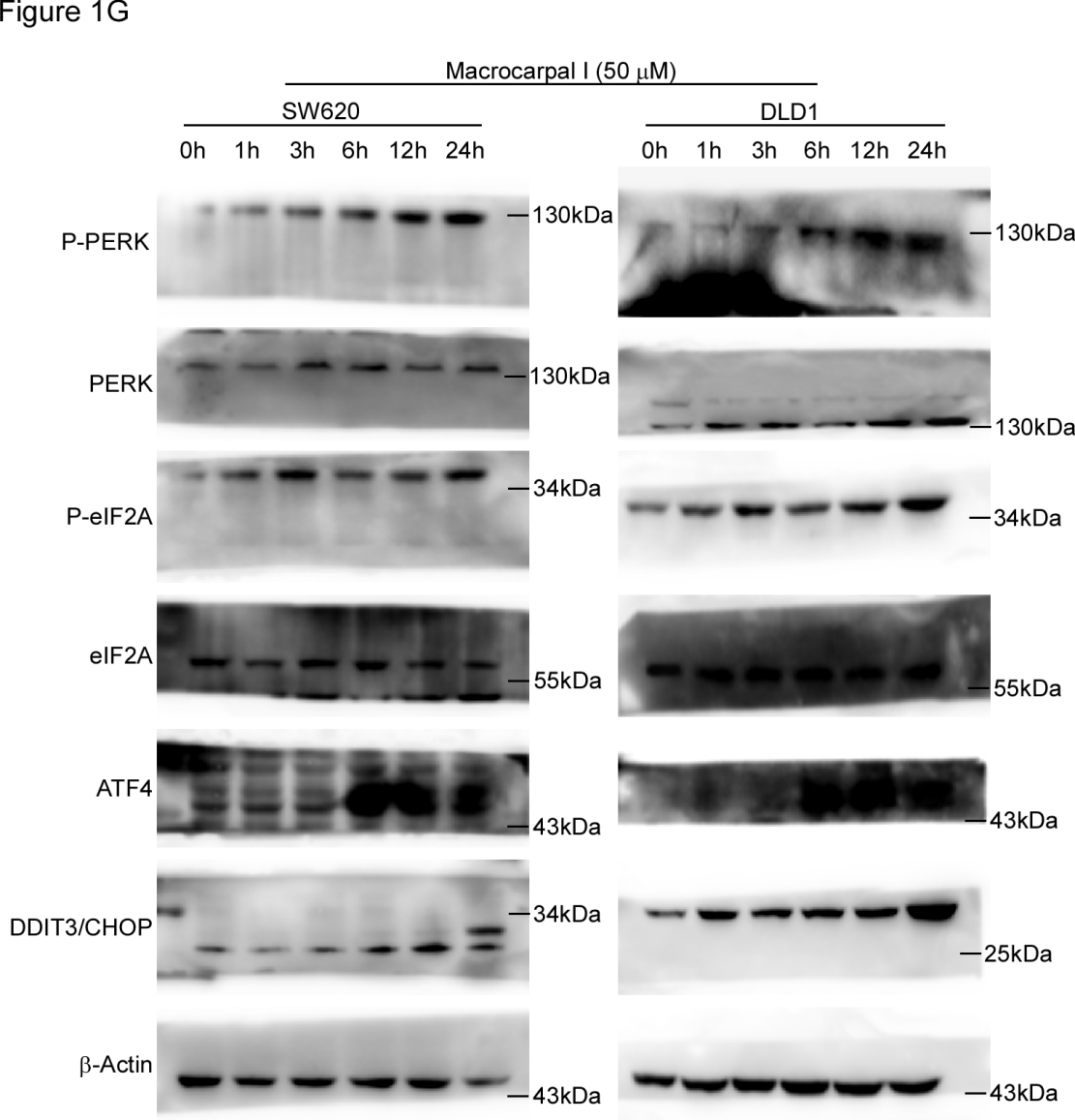


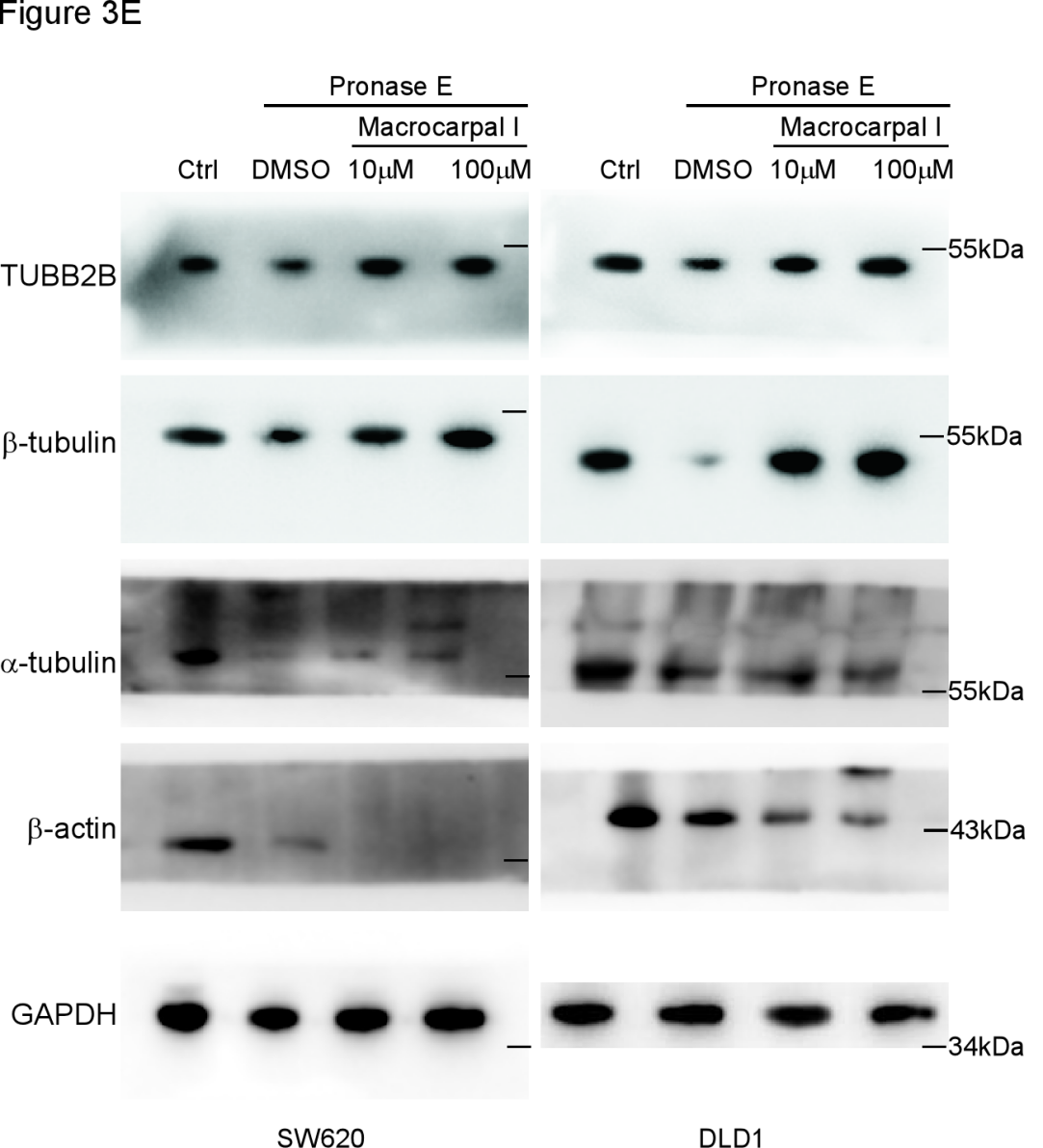


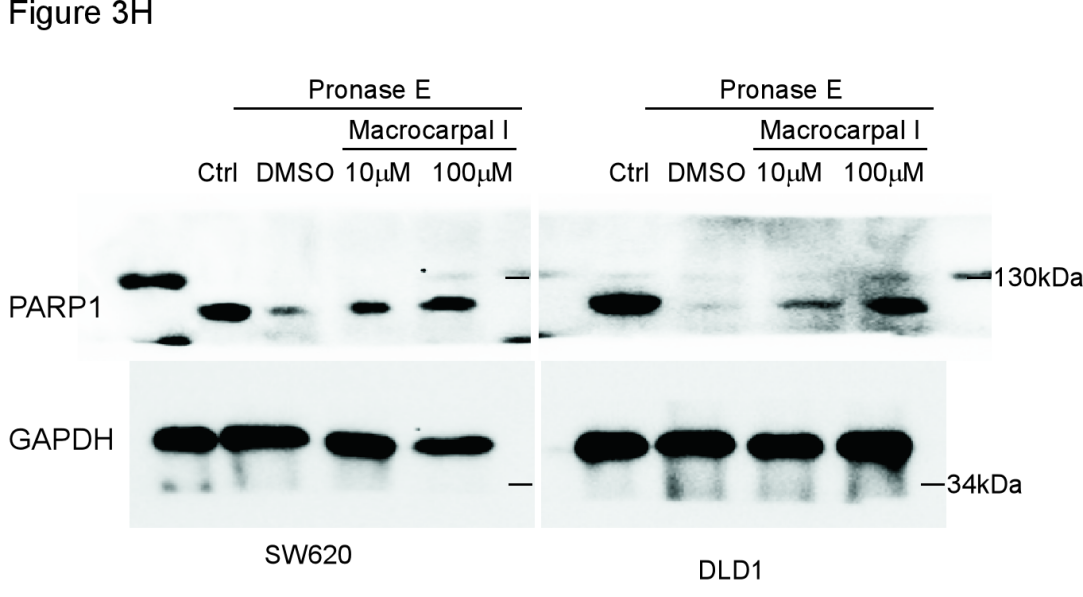


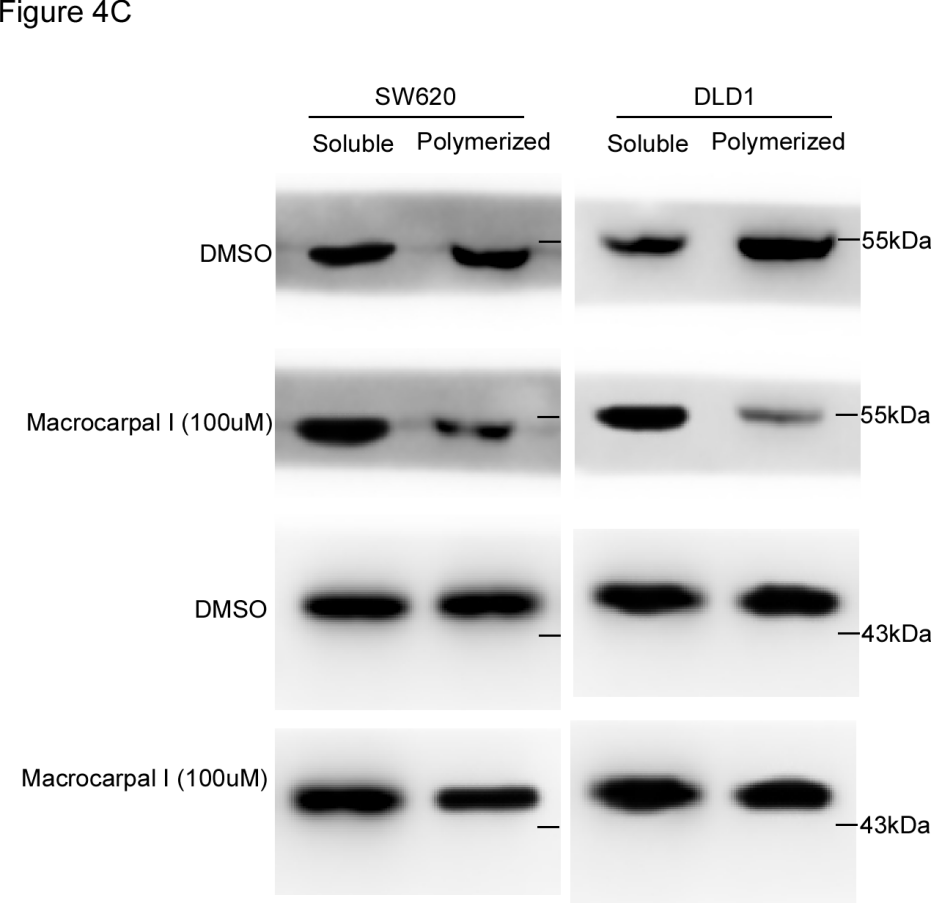


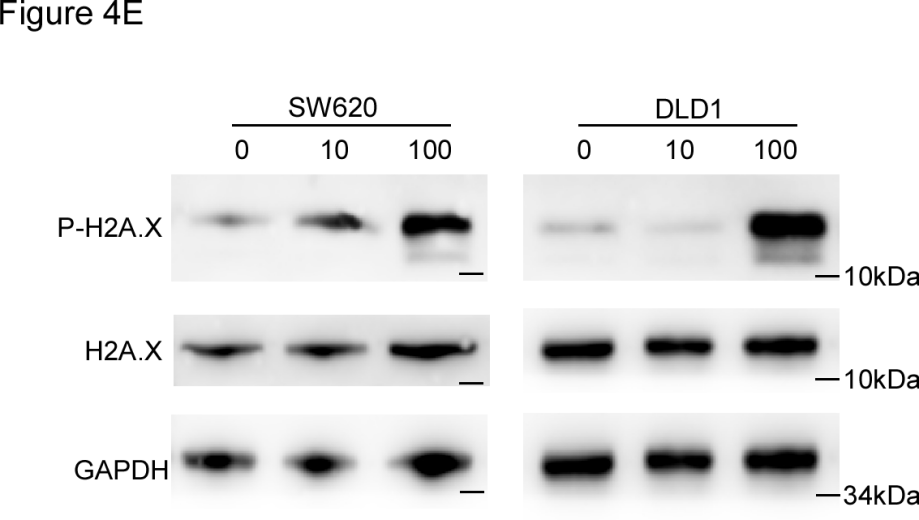


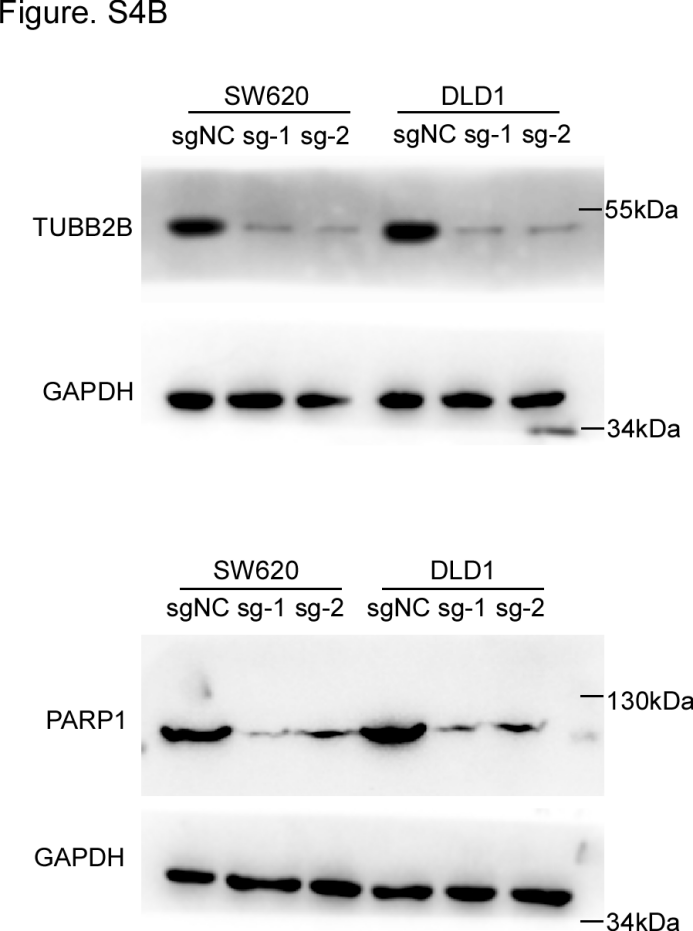

Supplement: Supplementary file 2 — Original western blot [file 41420_2025_2360_MOESM2_ESM.docx]
